# MnO_2_ Nanostructure-Based Novel Sensing: A Review

**DOI:** 10.3390/s26113544

**Published:** 2026-06-03

**Authors:** Haoyu Qi, Ting Ji, Fanjie Ji, Yan Wang

**Affiliations:** 1 School of Electronic Information, Northwest University, Xi’an 710127, China; 2 Xi’an Aerospace Composites Research Institute, Xi’an 710025, China; 3 School of Energy and Power Engineering, Huazhong University of Science and Technology, Wuhan 430074, China; 4 School of Chemical Engineering, Northwest University, Xi’an 710127, China

**Keywords:** MnO_2_ nanostructures, novel sensing, MnO_2_ quantum dot, 1D MnO_2_, 2D MnO_2_, hierarchical MnO_2_

## Abstract

Growing public concern over the living environment, food safety and the healthcare industry has spurred rapid advances in advanced sensing technology for environmental monitoring, food-safety screening, and biomedical surveillance. Consequently, developing a novel sensing strategy which is efficient, inexpensive, and easy to operate has become a major research focus in recent years. MnO_2_ nanostructures combine advantages of high specific surface area, quantum confinement, surface effects originating from their nanostructure, pronounced redox activity, broad optical absorption, distinctive electrochemical behavior, multiple accessible oxidation states, low cost and environmental benignity contributed by MnO_2_, which make them a critical material candidate for developing advanced sensing technology. This paper provides a comprehensive overview of MnO_2_ nanostructure-based novel sensors over the past five years. The contents of this review are listed as follows: (1) synthetic strategy and sensing advantages of MnO_2_ quantum dots, 1D MnO_2_, 2D MnO_2_ and hierarchical MnO_2_; (2) recent research advances in detection methodology and corresponding principles based on MnO_2_ nanostructures; and (3) the applicational progress of MnO_2_ nanostructure-based novel sensing technology in the field of food safety and biosensing. Finally, the foregoing discussion is integrated, and the current shortcomings and future development trends of novel sensors based on MnO_2_ nanostructures are critically assessed.

## 1. Introduction

Rapid modernization development has brought prosperity while also causing emerging social challenges, including environmental contamination, food-safety risk and the outbreak of infectious diseases. Due to the threat to air quality, water resources, food security and biosafety produced by airborne pollutants, including NH_3_, NO_2_, HCHO, CH_3_CHO and C_6_H_15_N [1]; heavy-metal ions, including Cr, As, Se, Sr, Hg, Pb, as well as N_2_H_4_·H_2_O; organophosphorus pesticides; antibiotic residue; and pathogens [2,3,4], it is urgently necessary to establish efficient and rapid analytical tools [5] for real-time and ultrasensitive monitoring of the above-mentioned contaminants [6].

Traditional strategies for pollutant detection include chromatography, atomic absorption spectrometry, atomic fluorescence spectrometry, microbial techniques and the enzyme-linked immunosorbent assay. Although it offers high response rates and resolutions, chromatography involves expensive equipment, complex operations and time-consuming procedures [7]. Spectrometry offers high efficiency and non-destructive detection, but suffers from limited selectivity and significant sensitivity variation [8,9]. The microbial technique is low-cost and environmentally friendly but exhibits slow responsiveness and poor stability [10]. The enzyme-linked immunosorbent assay boasts high specificity and response rates but relies heavily on antibodies and can only detect a single target [11]. Therefore, developing novel sensing technology with high sensitivity, high efficiency, excellent stability, low cost, a simple process and environmental friendliness would facilitate convenient, timely and precise monitoring of the target substance. In recent years, novel sensing technologies, consisting of electrochemistry, electrochemiluminescence (EL), photoelectron chemistry, colorimetry and fluorescence, have advanced rapidly [12,13,14,15,16,17,18]. For instance, the electrochemical sensor converts the components and concentration of the target into electrical signals including current, potential and conductivity *via* a redox reaction. The EL sensor identifies the target utilizing light signals generated from electrochemical reaction-excited luminescence, featuring high sensitivity and multi-component analyses.

Nanomaterials enhance the sensitivity of the sensor due to its high specific surface area, unique quantum confinement and surface effects, which shorten the diffusion path and improve the electron transport efficiency of the target. The size compatibility of nanomaterials enables the miniaturization and portability of sensors. Additionally, the high reactivity of nanomaterials enables a reduction in sensing costs. Most importantly, the outstanding photo-electro-thermal property of nanomaterials confers multimodal detection capabilities to the sensor to address numerous challenges in target analysis [19,20]. Manganese dioxide (MnO_2_) nanostructures have garnered significant attention due to their outstanding redox capability, broad optical absorption, unique electrochemical property, high charge transfer rate, diverse oxidation states, structure flexibility, large specific surface area, high physicochemical stability, environmental friendliness, as well as low cost [21,22]. Generally speaking, the MnO_2_ nanostructure is mainly applied in sensing in two ways. On the one hand, the MnO_2_ nanostructure is directly employed as the primary sensing material due to its unique physicochemical properties. On the other hand, due to its excellent carrier characteristics, MnO_2_ can be combined with other sensing materials to construct a novel sensor with multi-scenario adaptability.

Over the past five years, there have been numerous reports about the application of MnO_2_ nanostructures in sensing, yet comprehensive reviews in this area remain scarce [23,24,25]. In order to provide a comprehensive understanding of MnO_2_ nanostructure-based novel sensing, in this review, the synthesis method and sensing advantage of zero-dimensional (0D) MnO_2_ or MnO_2_ quantum dots, one-dimensional (1D) MnO_2_, two-dimensional (2D) MnO_2_ and hierarchical MnO_2_ are first summarized. Next, the detection principles and characteristics of MnO_2_ nanostructure-based novel sensors, including electrochemistry, EL, photoelectron chemistry, colorimetry, fluorescence, photothermal techniques and quartz microbalance (QCM), are systematically elucidated and compared. Most importantly, the specific application of MnO_2_ nanostructure-based sensors in environmental monitoring, food-safety monitoring and biomedical monitoring is comprehensively discussed. Finally, the existing limitations and future development trend of nano-MnO_2_-based sensors are briefly outlined.

## 2. Manganese Dioxide Nanostructure

**Scheme 1:**
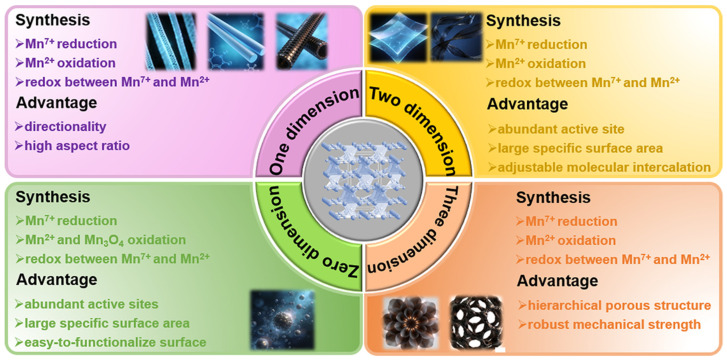
Synthesis strategy and sensing advantage of MnO_2_ nanostructure.

The sensing performance of MnO_2_ is highly dependent on its nanostructure, which in turn is closely related to the morphology and dimensionality of MnO_2_. MnO_2_ with different dimensions exhibits distinct advantages in sensing. The synthesis method and sensing advantage of 0D, 1D, 2D and three-dimensional (3D) MnO_2_ will be introduced and summarized systematically in the following (Scheme 1).

### 2.1. Zero-Dimensional MnO_2_

0D MnO_2_ refers to MnO_2_ materials with a size of three dimensions usually in the range of 1–100 nm and mainly including MnO_2_ nanoparticles and MnO_2_ quantum dots. In particular, the size of a MnO_2_ quantum dot in three dimensions is usually less than 10 nm, mostly concentrated in the range of 2–10 nm, which leads to strong constraints on carriers in three spatial directions and atomic-level discretization of electronic energy levels, finally contributing to significant quantum confinement effects. The most widely recognized MnO_2_ quantum dot owns intrinsic structural characteristics including an ultra-small size and a high surface-to-volume ratio, thereby possessing more edge active sites per unit mass. Moreover, due to the edge effect and quantum confinement, a MnO_2_ quantum dot exhibits enhanced or novel properties, such as high photoluminescence quantum efficiency. Furthermore, compared to the larger material, a MnO_2_ quantum dot exhibits optical stability, wavelength-dependent photoluminescence, chemical inertness, cellular permeability and biocompatibility, which make it a promising candidate in bio-related sensing technology. The strong light absorption ability makes 0D MnO_2_ nanomaterials promising energy acceptors in fluorescent sensors. The large specific surface area and outstanding catalytic property make 0D MnO_2_ nanomaterials substrates in electrochemical sensors. Furthermore, the high oxidative capacity of 0D MnO_2_ nanomaterials enables their widespread application in optical sensors.

The most common strategies for synthesizing 0D MnO_2_ include the reduction of KMnO_4_ by a reducing agent [26], the oxidation of MnCl_2_ by an oxidizing agent [27], the oxidation of Mn_3_O_4_ by an oxidizing agent [28], and a redox reaction between KMnO_4_ and Mn^2+^ [29]. The commonly used reducing agents for KMnO_4_ include metallic elements, sodium citrate, HCl, H_2_SO_4_, methionine, 2-(N-morpholino)ethanesulfonic acid, poly(2-hydroxypropyl)acrylate, poly(3-hydroxytyrosyl)acrylate, poly(allylamino)acrylate, glucose, acetone and casein. The commonly used oxidizing agents for MnCl_2_ include sodium hydroxide, H_2_O_2_ and bovine serum albumin. The oxidizing agent for Mn_3_O_4_ is NaOH. Common Mn^2+^ species involved in redox reactions with KMnO_4_ include MnCO_3_, MnCl_2_ and MnSO_4_.

**Figure 1:**
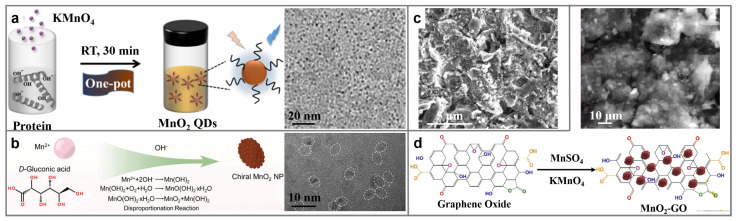
Synthesis and morphology of 1D MnO_2_. (**a**) Synthesis of CS-MnO_2_ QDs *via* reducing KMnO_4_ by *β*-casein and its TEM image; (**b**) synthesis of chiral MnO_2_ NPs *via* NaOH oxidation of MnCl_2_ and its TEM image; (**c**) scanning electron microscopy (SEM) images of MnO_2_/GO synthesized *via* NaOH oxidation of Mn_3_O_4_; (**d**) synthesis of MnO_2_ nanoparticle *via* redox reaction between KMnO_4_ and MnSO_4_ and its SEM image.

For biosensing application, Guo et al. synthesized *β*-casein–MnO_2_-quantum dots (CS-MnO_2_ QDs) *via* an in situ one-pot method at room temperature [30] (Figure 1a). In this system, the protein simultaneously functions as both a reducing agent and a stabilizer. Specifically, specific amino acid residue in casein reduces MnO_4_^−^ to MnO_2_, while the protein acts as a capping agent to control the growth and stabilize the morphology of the quantum dot. Compared to MnO_2_ quantum dots synthesized by a top–down strategy, this bottom–up approach is rapid, simple and environmentally friendly. The multifunctional CS-MnO_2_ QDs nanoenzyme exhibits superior catechol oxidase-like activity, enabling their application in fluorescence-enhanced biosensing of redox-active dopamine based on an interface passivation strategy. Kuang et al. reported the synthesis of chiral MnO_2_ nanoparticles (MnO_2_ NPs) using D-gluconic acid as a ligand *via* a disproportionation reaction [31]. In summary, as shown in Figure 1b, D-gluconic acid was pre-introduced into the MnCl_2_ solution to form Mn^2+^–gluconic acid complexes. Subsequently, Mn^2+^ was oxidized to Mn^4+^ in the presence of NaOH, which further induced the nucleation and growth of chiral MnO_2_ NPs. Transmission electron microscopy (TEM) images reveal that chiral MnO_2_ NPs exhibit uniform elliptical morphology with an average diameter of approximately 5 ± 0.8 nm. The designed chiral MnO_2_ NPs were applied for detecting intracellular H_2_O_2_ based on a dual-mode approach combining circular dichroism spectroscopy and magnetic resonance imaging. Eduard Llobet et al. synthesized a MnO_2_–graphene oxide composite (MnO_2_/GO) *via* the oxidation of Mn_3_O_4_ by NaOH [28] (Figure 1c). Briefly, graphene oxide and Mn_3_O_4_ were dispersed in oxalic acid. After thorough mixing, a NaOH solution was added to facilitate the gradual precipitation of MnO_2_ onto graphene oxide. Finally, MnO_2_/GO was obtained after filtering, drying and grinding. By controlling synthesis parameters such as temperature, stirring speed, addition rate and raw material ratio, a sample with desired crystallinity and optimal NO_2_ gas sensing performance was derived. Dinh Quang Khieu et al. synthesized MnO_2_ nanoparticles *via* a redox reaction between KMnO_4_ and MnSO_4_ [32]. The MnO_2_ nanoparticle was yielded by slowly adding a KMnO_4_ solution to a MnSO_4_ solution and further stirring at room temperature (Figure 1d). The specific reaction was as follows: 2KMnO_4_ + 3MnSO_4_ + 2H_2_O → 5MnO_2_ + K_2_SO_4_ + 2H_2_SO_4_. The synthesized MnO_2_ nanoparticle was applied to detecting biomolecules, including chloramphenicol and tinidazole, utilizing electrochemical technology.

### 2.2. One-Dimensional MnO_2_

1D MnO_2_ is characterized by nanoscale dimensions in one dimension, which commonly includes nanotubes, nanofibers, nanowires and nanorods. Due to significant elongation in a single direction, 1D MnO_2_ exhibits a rapid electron transport rate along the oriented axis, which further accelerates the response speed of the sensor. Furthermore, the interstitial space between 1D MnO_2_ chains promotes electrolyte diffusion, conferring significant sensing advantages in photocatalytic, electrocatalytic, and photoelectrocatalytic sensors [33]. Based on composition, 1D MnO_2_ can be categorized into single-component 1D MnO_2_ and 1D MnO_2_ composites. Single-component MnO_2_ offers advantages such as low cost and simple preparation, but its performance is limited. 1D MnO_2_ composites possess advantages of excellent conductivity, structural stability and large specific surface area due to synergistic effects, which expand their sensing applications in fields of biomedicine, environmental monitoring and remediation, as well as food-safety management.

Currently, the most prevalent strategies for synthesizing single-component 1D MnO_2_ include the reduction of KMnO_4_ using reducing agents [34], the oxidation of Mn^2+^ using oxidizing agents [35], and a redox reaction between KMnO_4_ and Mn^2+^ [36]. Ethanol, HCl, acetic acid, 2-morpholinoethanesulfonic acid, porous carbon, graphene oxide, polyaniline (PANI) and metal ions are often used for reducing KMnO_4_. The Mn^2+^ sources primarily include MnSO_4_ and MnAc_2_, and O_2_, NaOH and Na_2_SO_4_ are utilized to oxidize Mn^2+^. The Mn^2+^ involved in the redox reaction with KMnO_4_ is primarily MnSO_4_. There exist two primary strategies for preparing 1D MnO_2_ composites. One involves first synthesizing single-component 1D MnO_2_, and then other substances are assembled with it, or a small-sized target nanostructure is chemically modified onto it. Another involves directly growing single-component 1D MnO_2_ on a larger target composite or modifying the existing target single-component 1D MnO_2_ with the MnO_2_ nanostructure to form a heterostructure composite.

**Figure 2:**
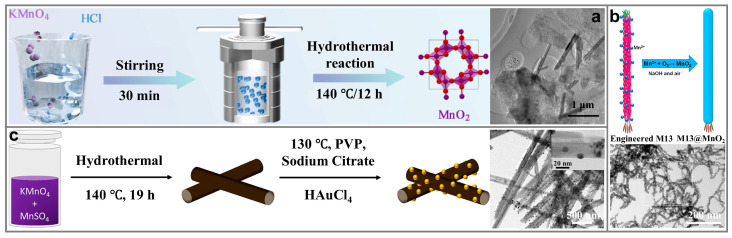
Synthesis and morphology of 1D MnO_2_. (**a**) TEM image of MnO_2_ hollow nanotube and ZnO nanosheet/MnO_2_ nanotube heterostructure synthesized *via* HCl reduction of KMnO_4_; (**b**) synthesis of M13-E4@MnO_2_ nanowire *via* NaOH oxidation of MnAc_2_ using soft M13 phage as template, along with its TEM image; (**c**) a TEM image of MnO_2_ nanowire and Au nanoparticle@MnO_2_ nanowire synthesized *via* redox reaction between KMnO_4_ and MnSO_4_.

Li et al. prepared a ZnO/MnO_2_ heterojunction nanocomposite through first synthesizing a 1D MnO_2_ nanotube followed by compositing it with a ZnO nanosheet. The KMnO_4_ was initially reduced by HCl to prepare the hollow MnO_2_ nanotube [37] (Figure 2a). Then, several ZnO/MnO_2_ binary hybrids were synthesized by incorporating varying amounts of the hollow MnO_2_ nanotube in the process of preparing the ZnO nanosheet. The thin ZnO nanosheet was uniformly modified with the hollow MnO_2_ nanotube, the wall thickness of which was approximately 40 nm. The ZnO/MnO_2_ heterostructure exhibits not only a large specific surface area and superior gas adsorption efficiency at the heterointerface but also enhanced charge carrier transport efficiency, resulting in significantly improved NH_3_ sensing performance under room temperature. Liu et al. prepared a 1D M13 phage@MnO_2_ (M13-E4@MnO_2_) nanowire at room temperature by adding NaOH to a mixed solution of M13-E4 and MnAc_2_ [38] (Figure 2b). Specifically, the carboxyl group bearing the E4 tag in M13-E4 first bound to Mn^2+^ through coordination interaction. Secondly, NaOH was added to a mixture containing M13 and Mn^2+^ in order to oxidize Mn^2+^ to MnO_2_ in air. In this process, O_2_ from the air and the weakly basic environment provided by NaOH are crucial. The MnO_2_-mineralized phage retains filamentous morphology, with a length of approximately 1 ± 0.1 μm and a diameter of 20–30 nm. The MnO_2_ crystal is uniformly distributed on the phage scaffold. The synthesized M13-E4@MnO_2_ nanowire can electrocatalytically oxidize H_2_O_2_ under neutral pH conditions, endowing significant potential for the biosensing of H_2_O_2_. Susana I. Córdoba de Torresi et al. directly modified a target Au nanoparticle onto a pre-synthesized 1D MnO_2_ nanomaterial, effectively enhancing the catalytic activity of 1D MnO_2_ and increasing the sensing sensitivity by 116%. First, a MnO_2_ nanowire was synthesized *via* a redox reaction between KMnO_4_ and MnSO_4_ [39] (Figure 2c). The synthesized MnO_2_ nanowire exhibits uniform morphology with a width of 50 ± 10 nm and a length of 1 μm, approximately. Subsequently, the nanowire served as rigid template for the uniform nucleation and growth of Au nanoparticles. The incorporated Au nanoparticle enhances the photocatalytic performance of the MnO_2_ nanowire through a localized surface plasmon resonance effect. This composite material was applied to enhance H_2_O_2_ sensing based on photocatalytic oxidation.

### 2.3. Two-Dimensional MnO_2_

A 2D nanostructure is marked by nanoscale dimensions in two dimensions, such as nanoscale monolayers and multilayers, nanoflakes and nanoribbons. 2D MnO_2_ primarily encompasses MnO_2_ nanoflakes and related composites. Similar to 1D MnO_2_, single-component 2D MnO_2_ is readily synthesized *via* a simple process, while exhibiting limited applicability and sensing performance. When MnO_2_ is composited with other sensing substrates, the sensing performance, including responsiveness and repeatability, can be significantly enhanced.

The most prevalent strategies for synthesizing 2D MnO_2_ include the reduction of KMnO_4_ using a reducing agent [33], the oxidation of Mn^2+^ using an oxidizing agent [40], and a redox reaction between KMnO_4_ and Mn^2+^ [41]. Reduction agents such as ethanol, HCl, H_2_SO_4_, 2-(N-morpholino)ethanesulfonic acid, 3-morpholinopropanesulfonic acid, ethyl acetate, metallic elements and metal hydroxide are usually utilized to reduce KMnO_4_. H_2_O_2_, NaOH and triethanolamine are commonly used oxidizing agents for Mn^2+^. The Mn^2+^ used to reduce KMnO_4_ is mainly MnCl_2_, MnSO_4_ and MnAc_2_.

**Figure 3:**
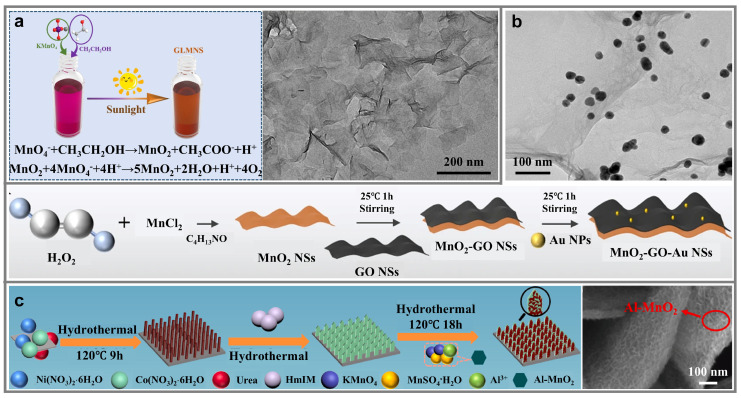
Synthesis and morphology of 2D MnO_2_. (**a**) Synthesis of carbonaceous *δ*–MnO_2_ nanosheet *via* CH_3_CH_2_OH reduction of KMnO_4_ under solar light irradiation and its TEM image; (**b**) synthesis of 2D MnO_2_ nanosheet@graphene oxide layer@Au nanoparticle composite *via* H_2_O_2_ oxidation of MnCl_2_ and its TEM image; (**c**) synthesis of 2D MnO_2_ nanosheet and layered Ni-Co double metal hydroxide@Al-MnO_2_ nanosheet *via* redox reaction between KMnO_4_ and MnSO_4_, with corresponding SEM image.

Xiong et al. proposed a top–down solar-assisted green strategy for preparing 2D MnO_2_, which is environmentally friendly, simple, low-cost and rapid [42]. As shown in Figure 3a, ethanol was first added to a KMnO_4_ solution as the electron acceptor and mixed thoroughly at room temperature. The precursor solution was then placed in a quartz flask and exposed to sunlight to initiate the reaction. During the process, the solution color changed from purple–red to dark brown. TEM images reveal that the synthesized MnO_2_ nanosheet exhibits an ultrathin, nearly transparent 2D-layered structure with abundant graphene-like wrinkles. The synthesized MnO_2_ nanosheet demonstrates excellent dispersion in aqueous solution without a surface stabilizer, facilitating its convenient application in colorimetric sensing. The synthesized MnO_2_ nanosheet demonstrated rapid, sensitive colorimetric sensing for detecting triclosan in wastewater and assessing total antioxidant capacity in human serum. To enhance detection specificity and the high sensitivity demanded in biosensing, single-component MnO_2_ is often limited. Conversely, integrating multiple sensing substrates and achieving synergistic enhancement between them enables the preparation of a biosensor with higher catalytic activity and selectivity, as well as chemical stability. In this article, a 2D MnO_2_ nanosheet was synthesized *via* a one-step oxidation reaction of MnCl_2_ by H_2_O_2_ [43]. Specifically, MnCl_2_ was first added to a solution containing tetramethylammonium hydroxide and H_2_O_2_ and was stirred overnight. Then, the MnO_2_ nanosheet was prepared through centrifugation and washing (Figure 3b). SEM images reveal that the product consists of wrinkled, ultrathin, monolayer nanosheets. Finally, the synthesized MnO_2_ nanosheet@graphene oxide layer@Au nanoparticle composite was utilized to construct a novel electrochemistry-based cell sensor for detecting CTC in blood, demonstrating specific selectivity and sensitivity with broad application prospects in clinical diagnostics. Based on a redox reaction between KMnO_4_ and MnSO_4_, a 2D Al-MnO_2_ nanosheet was first synthesized *via* a one-step hydrothermal method by Liu et al. The final composite was produced by combining the 2D Al-MnO_2_ nanosheet with a NiCo-MOF nanowire array [44] (Figure 3c). SEM images reveal that the synthesized MnO_2_ is composed of multiple nanosheets, each approximately 20 nm thick and 200 nm in diameter. Given the inherently poor conductivity of pure MnO_2_, Al doping effectively narrows the band gap and increases the electron concentration in the conduction band of pure MnO_2_, which further enhances the charge transport capability of pure MnO_2_. It is worth noting that the electrostatic repulsion between negatively charged manganese vacancies within the MnO_2_ nanosheet maintains the structural stability of nanosheet colloids, further improving the performance of fluorescence sensing [45,46,47].

A 2D MnO_2_ nanosheet has great potential in sensing due to its large specific surface area. However, the practical application of 2D MnO_2_ still faces the challenges of insufficient long-term stability and poor reusability. On the one hand, when there exists a redox reaction between MnO_2_ and target analytes such as glutathione and H_2_O_2_, Mn^4+^ will be reduced to soluble Mn^2+^, which not only leads to irreversible consumption of the active substrate but also causes the collapse of the lamellar structure in MnO_2_, eventually leading to the loss of reactive sites. During analyzing serum and environmental water samples, biomacromolecules such as protein can be irreversibly adsorbed on the surface of a MnO_2_ nanosheet to form a pollution layer, which hinders the adsorption of the target and electron transfer across the substrate. Meanwhile, due to its high surface energy, a 2D nanosheet is prone to stacking and agglomeration during the period of long-term storage and repeated usage, resulting in a decrease in the effective specific surface area and the number of active sites. In order to solve the above-mentioned challenges, there exist several strategies, including element doping, heterostructure construction and compositing with the second material, which can effectively improve the structural stability of 2D MnO_2_ nanosheets.

### 2.4. Three-Dimensional MnO_2_

3D hierarchical structures are assembled by diverse structural units consisting of 0D, 1D or 2D nanostructures. Common 3D hierarchical structures include nanoclusters or nanoflowers and spherical or box-shaped hollow mesoporous nanomaterials. A 3D nanostructure effectively increases the specific surface area of the material, providing more active sites for sensing. In addition, a 3D nanostructure allows for the adsorption and reaction of more target molecules, thereby enhancing sensing sensitivity. The large specific surface area and reduced transfer resistance for ions and electrons of 3D MnO_2_ make it suitable for (photo)electrochemical sensors. Furthermore, 3D structures exhibit high mechanical strength, rendering them suitable for sensing under extreme conditions, such as ultra-high or ultra-low temperature and ultra-high or ultra-low pressure [21,48,49,50,51,52].

The reduction of KMnO_4_ using a reducing agent [53], the oxidation of Mn^2+^ using an oxidizing agent [54], and a redox reaction between KMnO_4_ and Mn^2+^ [18] are often utilized to prepare 3D MnO_2_. There exist various reduction agents for KMnO_4_ reduction, such as HCl, 2-(N-morpholino) ethanesulfonic acid, oleic acid, polyallylamine hydrochloride, graphite and carbon fiber. The Mn^2+^ sources primarily include MnCl_2_, MnSO_4_ and MnAc_2_. H_2_O_2_, Na_2_SO_4_, K_2_S_2_O_8_, (NH_4_)_2_S_2_O_8_ and K_3_[Fe(CN)_6_] are mainly utilized to oxidize Mn^2+^. Common Mn^2+^ sources involved in redox reactions with KMnO_4_ include MnCl_2_, MnCO_3_ and MnSO_4_.

**Figure 4:**
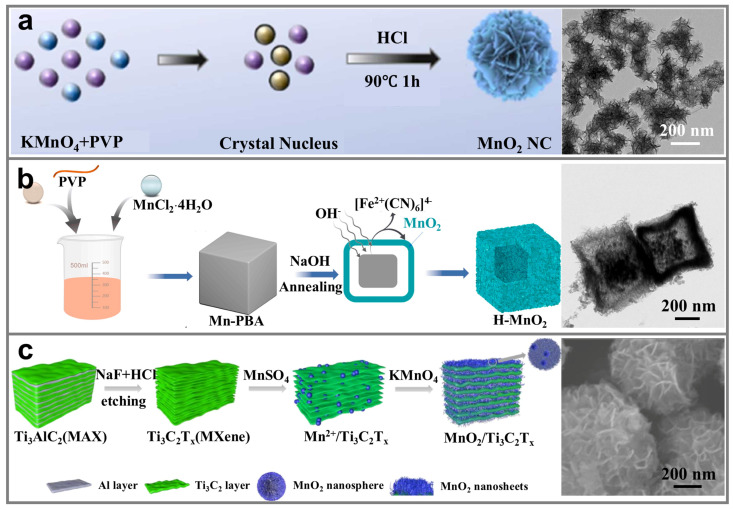
Synthesis and morphology of 3D MnO_2_. (**a**) Synthesis of MnO_2_ nanocluster using polyvinylpyrrolidone as surfactant *via* HCl reduction of KMnO_4_, along with its TEM image; (**b**) synthesis of MnO_2_ hollow cube *via* in situ oxidation of MnCl_2_·4H_2_O by K_3_Fe(CN)_6_ and its TEM image; (**c**) synthesis of MnO_2_ nanoblossom *via* redox reaction between KMnO_4_ and MnSO_4_ and its SEM image.

By utilizing a smartphone app platform and employing a 3D MnO_2_ nanocluster as the signal probe to effectively amplify biological signals, Lin et al. not only ensured specific color assessment in colorimetric detection but also achieved real-time synchronous monitoring of Salmonella by coupling sampling time with sampling location. The 3D MnO_2_ nanocluster was synthesized by reducing KMnO_4_, using polyvinylpyrrolidone as the surfactant and HCl as the reducing agent respectively [55] (Figure 4a). Considering the potential risks associated with the hydrothermal method and the utilization of the strong oxidizing agent KMnO_4_, Yue et al. employed a self-templating method to synthesize hollow cubic MnO_2_ via an in situ oxidizing reaction [56] (Figure 4b). The resulting nanosheet and hollow hierarchical structure not only exposes more active sites to enhance reaction activity but also improves electron transfer kinetics and accelerates the reaction rate. Moreover, compared to the conventional bulk structure, the hollow nanostructure effectively releases stress generated during the sensing reaction, thereby enhancing its long-term operational stability. Additionally, the generated oxygen vacancy can serve as an active site for oxygen adsorption, which promotes the catalytic reaction. A more detailed explanation of how these vacancies are intentionally characterized is necessary. The 530.4 and 531.5 eV peaks in the O 1s spectrum are attributed to Mn-OH and H-O-H, respectively, which are caused by oxygen defects, indicating the presence of oxygen vacancies in H-MnO_2_. The electron paramagnetic resonance (EPR) signal at g = 2.003 further demonstrates that H-MnO_2_ has oxygen vacancies. The specific synthesis process is as follows: a surface-smooth cubic manganese complex intermediate was first synthesized *via* a co-precipitation strategy based on an oxidation reaction between K_3_[Fe(CN)_6_] and MnCl_2_·4H_2_O; subsequently, ferricyanide ions and Mn^2+^ diffused to the surface of the cubic template of the complex and formed the hollow MnO_2_ structure by an in situ redox reaction with OH^−^. TEM images reveal that the MnO_2_ hollow cube not only retains its original cubic structure but also exhibits a hollow configuration. This hollow structure, composed of numerous nanosheets, endows the material with a large specific surface area. The authors developed a smartphone-assisted portable colorimetric sensor by encapsulating the MnO_2_ hollow cube in sodium alginate for on-site, real-time, specific and rapid colorimetric detection of three liver function biomarkers, including aspartate aminotransferase, alanine aminotransferase and alkaline phosphatase. The 3D heterojunction material can be obtained by growing the MnO_2_ nanostructure on the existing 3D framework. The strong interfacial interaction at the heterojunction enhances sensitivity, reactivity and stability of the sensing material. Zhu et al. synthesized a MnO_2_ nanoblock *via* a one-step hydrothermal method based on a redox reaction between KMnO_4_ and MnSO_4_ [57] (Figure 4c). SEM images reveal that the synthesized MnO_2_ nano-blossom consists of small-sized MnO_2_ nanosheets, with an average diameter of approximately 300–400 nm. Due to the excellent catalytic activity, the presence of abundant oxygen vacancies and *p-p* heterojunction of the obtained MnO_2_/titanium carbide composite, it was applied to detect volatile organic compounds such as hexanal, a lung cancer biomarker, demonstrating good reproducibility and stability.

The performance differences of MnO_2_ nanostructures with different dimensions (0D, 1D, 2D, and 3D) in electrochemical sensing are primarily manifested in four aspects: effective surface area, electron transfer pathways, analyte diffusion efficiency, and accessibility of active sites.

0D MnO_2_ boasts an extremely high specific surface area, offering abundant adsorption sites. However, it suffers from high inter-particle contact resistance, impeding electron hopping transfer between particles and leading to a high overall charge transfer resistance (R_ct_). Additionally, it tends to agglomerate, compromising its long-term stability. 1D MnO_2_ offers several advantages in which electrons can rapidly conduct along the long axis of nanowires, significantly reducing R_ct_. Intertwined into a 3D network, it provides continuous electrolyte diffusion channels. Yet, its specific surface area is slightly lower, with active sites predominantly distributed on the side and end faces. 2D MnO_2_ possesses superior sensing capabilities, and its ultra-high theoretical specific surface area and exposed active edge sites facilitate proton intercalation/deintercalation by allowing for the insertion of ions or water molecules between layers. The abundant -OH on its surface enhances hydrogen bonding/electrostatic interactions with analytes. However, nanosheets are prone to stacking, leading to poor electron conduction perpendicular to the layer direction. 3D MnO_2_ combines the high activity of low-dimensional units with the mechanical stability of the overall structure. The coexistence of macropores and mesopores promotes rapid electrolyte infiltration and product release. However, some active sites within 3D MnO_2_ are buried, resulting in a utilization rate that is lower than theoretical values.

In summary, the electrochemical sensing performance of MnO_2_ nanostructures with different dimensions is determined by their electron conduction pathways, effective surface area, and accessibility of active sites. 1D MnO_2_ achieves an optimal balance between sensitivity and stability due to its quasi-1D electron channel and good dispersibility. 2D MnO_2_ provides the highest response current per unit area and the lowest oxidation overpotential, making it suitable for the high-sensitivity detection of molecules such as neurotransmitters. 3D MnO_2_ exhibits the best mass transfer capability and pre-enrichment effect, achieving the lowest detection limit in the detection of trace heavy metals and glucose. Although 0D MnO_2_ is easy to synthesize, it is limited by inter-particle resistance and agglomeration issues, making it suitable only for scenarios where sensitivity is not critical.

## 3. Manganese Dioxide Polymorph

MnO_2_ consists of several crystal structures, containing mainly *α*-, *β*-, *γ*-, *δ*-, *ε*-, *λ*- and R-forms, which are formed *via* the common-edge or common-angle connection of octahedron MnO_6_ units. Among them, *α*-, *β*-, and *γ*-MnO_2_ belong to chain tunnel structures. Specifically, *α*-MnO_2_ is characterized by a “2 × 2, ~0.46 nm × 0.46 nm” tunnel structure. *β*-MnO_2_ has a “1 × 1, ~0.23 nm × 0.23 nm” tunnel structure with a tunnel size of 2.3 × 2 Å. *γ*-MnO_2_ is a rhombohedral crystal system composed of alternating “1 × 1, ~0.23 nm × 0.23 nm” channels of pyrolusite and “1 × 2, ~0.23 nm × 0.46 nm” channels of rhodochrosite. *δ*-MnO_2_ exhibits a representative 2D laminar structure characterized by a substantial inter-lamellar spacing of approximately 7 Å. The crystal structure of *ε*-MnO_2_ is relatively disordered. *λ*-MnO_2_ has a 3D structure constructed by arranging the tunnel structures in different ways. *R*-MnO_2_ has a long-range disordered structure.

MnO_2_ with different crystal phases has different semiconductor properties due to the different arrangement of atoms. *α*-MnO_2_ belongs to *n*-type semiconductors, which has a band gap of 1.3–2.7 eV and high conductivity of about 5.98 cm^−1^·Ω^−1^. *β*-MnO_2_ belongs to *n*-type semiconductors, which has a narrow band gap of 0.2–0.37 eV and a conductivity of about 1.74 × 10^−3^ cm^−1^·Ω^−1^. *γ*-MnO_2_ belongs to *n*-type semiconductors, and its conductivity is lower than that of *β*-MnO_2_. *δ*-MnO_2_, which belongs to *n*-type semiconductors, has a band gap of 1.36–2.0 eV and poor conductivity. The carrier type of *ε*- and *λ*-MnO_2_ is not clear. The carrier type of *R*-MnO_2_ is mainly *n*-type and has a band gap of 2.06–2.52 eV and poor conductivity.

As is known to all, the electrocatalytic activity and electrochemical sensing performance of MnO_2_ are closely dependent on its crystal structure. Due to fully exposed MnO_6_ edges and abundant oxygen vacancies contributed by the long Mn-O bond and low average oxidation state of Mn, *α*-MnO_2_ has a high specific surface area and excellent electrocatalytic activity. Due to the narrow tunnel of the *β*-MnO_2_-contributed molecular size screening effect, only small molecules can reach the active site, and macromolecules are selectively excluded. Although its electrocatalytic activity is usually lower than that of *α*-MnO_2_ and *β*-MnO_2_, *γ*-MnO_2_ has the advantage of surface chemical adjustability. The layered structure of *δ*-MnO_2_ endows it with the maximum available area and abundant oxygen vacancies, and the interlayer water in it contributes to ion transport. The crystal structure of *ε*-MnO_2_ is relatively disordered, and its specific surface area and specific capacitance are low. The 3D spinel tunnel interconnection structure of *λ*-MnO_2_ can accommodate a variety of ions without significant volumetric change, and there is little research on applying *λ*-MnO_2_ in sensing. The long-range disordered structure of *R*-MnO_2_ gives it a large number of defects and dangling bonds as active sites, contributing to particularly high surface activity.

It is necessary to discuss the mixed valence states of MnO_2_ and their impact on sensing performance. Firstly, the distribution of manganese oxidation states in different crystal forms of MnO_2_ should be discussed. The basic structural unit of MnO_2_ is the [MnO_6_] octahedron, and theoretically, Mn has a valence of +4. However, due to oxygen vacancies, proton insertion, or the insertion of alkali metal ions during crystal growth, some Mn^4+^ is reduced to Mn^3+^ to maintain charge balance. The proportion of Mn^3+^ and its stability vary significantly among different crystal forms. In *α*-MnO_2_, when the tunnel contains large-sized cations, it can stabilize a large amount of Mn^3+^, with a molar ratio of Mn^3+^/(Mn^4+^ + Mn^3+^), reaching 15–30%. These Mn^3+^ are located near the vertices of the octahedra, adjacent to oxygen vacancies, forming localized electronic states. In *δ*-MnO_2_, there are usually water molecules and cations between the layers, with a high proportion of Mn^3+^ (10–40%), which varies with the type of cations between the layers. The presence of Mn^3+^ leads to an increase in the interlayer spacing and an improvement in electronic conductivity. In *β*-MnO_2_, it has a dense structure and contains almost no tunnel cations, with the proportion of Mn^3+^ typically being less than 5%, exhibiting near-stoichiometric Mn^4+^. In *γ*-MnO_2_, it contains a large number of structural defects and Mn^3+^ (20–35%) and is often used as a battery cathode material, but it is less commonly used in sensing applications. Most importantly, the mechanism of the influence of the oxidation state on electrochemical sensing performance should be demonstrated. The oxidation state of Mn in MnO_2_ could influence the performance of sensors, including charge transfer and conductivity, catalytic active center and analyte selectivity. Just in terms of charge transfer and conductivity, the 3*d* electronic configuration of Mn^3+^ is t_2_g^3^ e_g^1^, which exhibits a higher tendency for electron localization compared to Mn^4+^ (t_2_g^3^ e_g^0^). However, the double exchange interaction between Mn^3+^-O-Mn^4+^ can generate mixed-valence conductive channels. Therefore, an appropriate amount of Mn^3+^ can significantly reduce the charge transfer resistance (R_ct_) of the material. For example, *δ*-MnO_2_ nanosheets rich in Mn^3+^ exhibit an R_ct_ for H_2_O_2_ that is 1–2 orders of magnitude lower than that of *β*-MnO_2_. As for the catalytic active center, the Mn^3+^ site is considered to be the true active center for many electrocatalytic reactions, such as H_2_O_2_ reduction, glucose oxidation, and ascorbic acid oxidation. Mechanistically, Mn^3+^ is more prone to bind with dissolved oxygen or peroxides, forming highly active Mn^3+^-(O_2_) or Mn^3+^-(OH) intermediates, which subsequently complete the catalytic cycle through electron transfer from Mn^3+^ to Mn^4+^. An excessively high proportion of Mn^3+^ may lead to aggregation of Mn^3+^ sites, forming an inert Mn_2_O_3_ phase, which in turn reduces catalytic activity. Therefore, there exists an optimal Mn^3+^/(Mn^4+^ + Mn^3+^) ratio to achieve the maximum response current. Moreover, oxidation states of Mn will influence the analyte selectivity in sensing. Different analytes exhibit varying affinities for the oxidation states of manganese. For example, H_2_O_2_ tends to coordinate with Mn^4+^, subsequently accepting electrons to generate •OH and OH^−^, with Mn^4+^ being reduced to Mn^3+^. Therefore, materials with a high initial proportion of Mn^3+^ can complete this reduction step more quickly, whereas dopamine preferentially complexes with surface Mn^3+^ and undergoes oxidation through ligand-to-metal charge transfer (LMCT). The response of dopamine to *δ*-MnO_2_, which is rich in Mn^3+^, is typically 5–10 times greater than that to *β*-MnO_2_.

## 4. Manganese Dioxide Nanostructure-Based Novel Sensing Technology

**Scheme 2:**
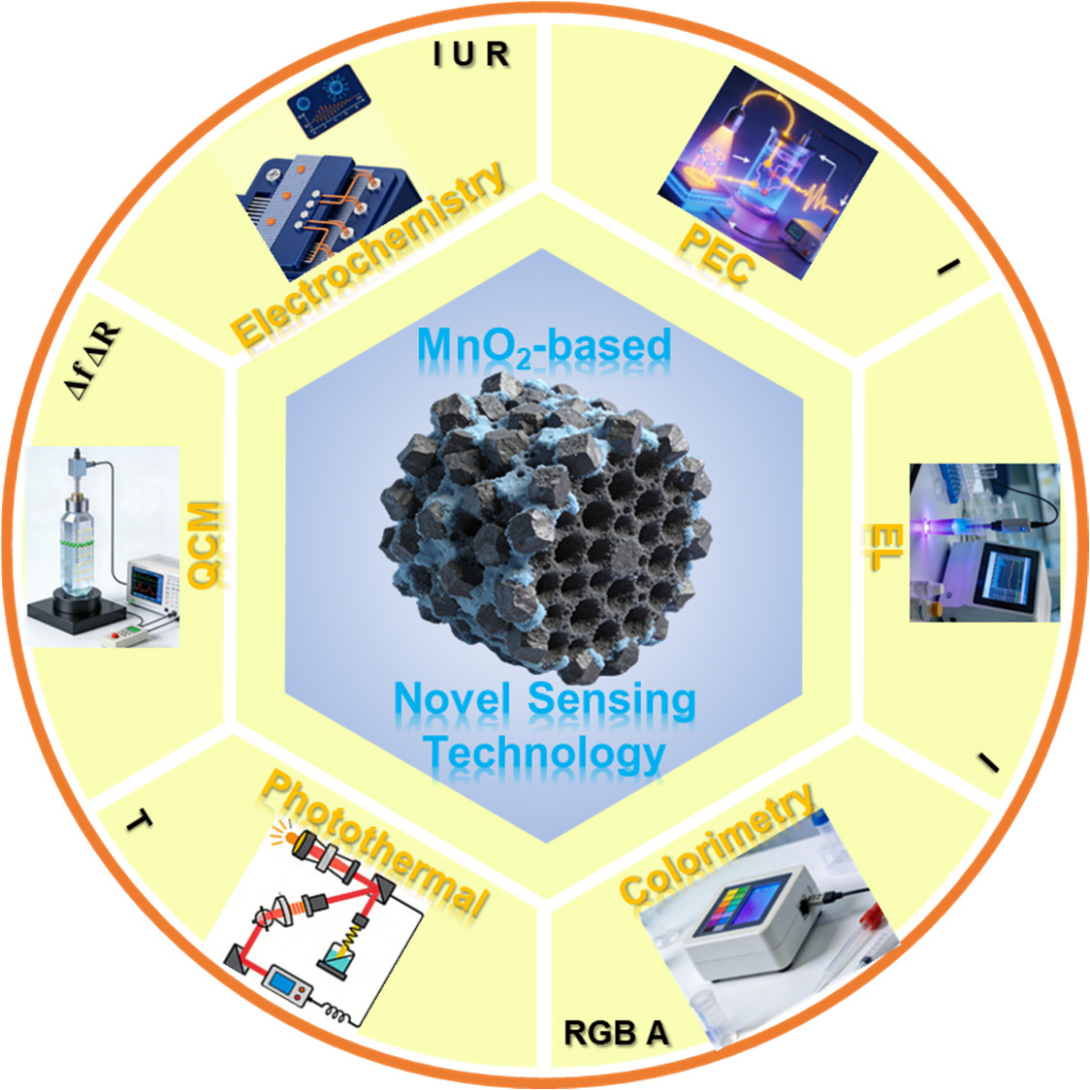
MnO_2_ nanostructure-based novel sensing technology.

Chemical sensors can be categorized into chemical reaction-based and physical interaction-based types, depending on the interactions between the receptor and the target substance [58]. In the process of chemical reaction-based sensing, a chemical reaction between the target and the receptor occurs, such as redox. Novel chemical reaction-based sensors include electrochemical sensors, EL sensors, photoelectrochemical (PEC) sensors and colorimetric sensors. Physical interaction-based sensing involves highly specific physical interaction between the target and the receptor. Novel physical interaction-based sensors include fluorescence sensors, photothermal sensors and QCM sensors (Scheme 2). MnO_2_ represents one of the ideal candidate materials for constructing a high-performance novel sensor. However, pure MnO_2_ suffers from poor conductivity and structural instability. Therefore, compositing MnO_2_ with other functional materials not only compensates for its inherent disadvantage but also achieves a synergistic enhancement effect.

### 4.1. Electrochemical Sensor

An electrochemical sensor conducts analyte identification by converting the component and concentration of the target into a current, potential or conductivity signal through a redox reaction between the target analyte and the recognition layer [59]. To date, electrochemical sensors have become the most widely applied type due to their high sensitivity, high efficiency, low cost, low detection limit and versatile signal consisting of voltage, current and impedance. As mentioned above, MnO_2_ possesses flexible valence states that can reduce overpotential in electrochemical reactions and enhance the current response in the system, thereby improving the sensitivity and selectivity of electrochemical sensors. Simultaneously, the high specific surface area of the MnO_2_ nanostructure provides abundant active sites for adsorbing and reacting with target analytes, enabling faster mass transport and stronger signal output within the system, thereby further improving the sensitivity and response speed of electrochemical sensors. However, pure MnO_2_ applied in electrochemical sensors suffers from poor conductivity, whereas a synergistic enhancement effect can be achieved by combining pure MnO_2_ with other highly conductive materials. The incorporation of highly conductive materials, like graphenes, carbon materials and conductive polymers, not only enhances the electron transport rate within MnO_2_ but also effectively prevents agglomeration of MnO_2_ nanoparticles, which contributes to the full exposure of active sites, thereby further improving the signal strength and sensitivity of electrochemical sensors. Noble metal nanoparticles like Au, Ag, Pt and Pd inherently possess excellent conductivity and exhibit catalytic activity toward numerous reactions, which not only improve electron transport efficiency within MnO_2_ but also generate dual-enhancement catalytic effects with MnO_2_, thereby enhancing the selectivity and sensitivity of electrochemical sensing. Consequently, the MnO_2_ nanostructure and its composites offer exceptionally prominent advantages when applied in novel electrochemical sensors.

A more in-depth discussion of the interactions between MnO_2_ and target analytes, charge transfer and catalytic activity is essential for a comprehensive review on MnO_2_-based electrochemical sensors.

A deep understanding of the chemical interaction between MnO_2_ and the analyte is a prerequisite for the rational design of highly selective sensors. Depending on the analyte, the interaction mechanism can be divided into three categories: electrostatic interactions and hydrogen bonding, enzyme-like catalytic oxidation, ion exchange and surface complexation. Electrostatic interactions and hydrogen bonding are first discussed. Under neutral pH conditions, the isoelectric point of MnO_2_ is approximately 2–4, indicating a negatively charged surface. Dopamine undergoes protonation of its amine group at physiological pH, resulting in a positively charged state, thus generating a strong electrostatic attraction with MnO_2_. Simultaneously, the catechol group of dopamine can form a bidentate hydrogen bond with the hydroxyl group on the surface of MnO_2_. This pre-adsorption significantly reduces the distance between the active site and the analyte, enabling rapid electron transfer to the Mn^4+^ center *via* a tunneling effect. Subsequently, dopamine is oxidized to dopamine quinone, releasing two electrons and two protons, which are transferred to the electrode through the aforementioned Mn^3+^/Mn^4+^ pathway. Enzyme-like catalytic oxidation is then demonstrated. MnO_2_ exhibits efficient catalase-like activity. Its catalytic cycle can be summarized as follows: Mn^4+^ + H_2_O_2_ → Mn^3+^ + •OOH + H^+^, Mn^3+^ + H_2_O_2_ → Mn^4+^ + •OH + OH^−^. The generated free radicals exhibit strong oxidizing properties and can further oxidize other substrates on the electrode surface. This cascade reaction renders MnO_2_ an ideal material for enzyme-free sensors, circumventing the issue of natural enzyme deactivation. It is necessary to illustrate ion exchange and surface complexation. The K^+^ or Na^+^ ions between the layers of layered *δ*-MnO_2_ can undergo ion exchange with heavy metal ions in solution. Meanwhile, the vacant sites in the tunnel structure can form inner-sphere complexes with heavy metal ions. In anodic stripping voltammetry (ASV) detection, after the enrichment step, applying a negative potential reduces the heavy metal ions to zero valence and deposits them on the surface of MnO_2_; then, applying a forward scan, the metals are re-stripped as ions, generating sharp current peaks. The tunnel size of MnO_2_ determines the ion selectivity: 2 × 2 tunnels (*α*-MnO_2_) have a preferential embedding ability for Pb^2+^, while 1 × 1 tunnels (*β*-MnO_2_) essentially exclude Pb^2+^.

The electrochemical sensing performance of MnO_2_ is essentially determined by the efficiency of heterogeneous electron transfer (HET) between the analyte and the electrode interface. This process is regulated by two key factors: the crystal structure and surface electronic density of states of MnO_2_. Different crystal forms of MnO_2_ (*α*, *β*, *γ*, *δ*, and *λ*) exhibit distinct [MnO_6_] octahedral connection modes, forming tunnel or layered structures. Among them, *δ*-MnO_2_ and *α*-MnO_2_ demonstrate the lowest charge transfer resistance (R_ct_). This is attributed to the presence of numerous Mn^3+^ sites and oxygen vacancies in their structures, which significantly enhance the density of electronic states near the Fermi level. In contrast, *β*-MnO_2_ exhibits near-insulating semiconductor behavior due to the extremely low proportion of Mn^3+^, leading to a sluggish HET process. More importantly, the surface redox couple of Mn^3+^/Mn^4+^ acts as an intermediate energy level for “electron hopping”. When an appropriate potential is applied, the Mn^4+^ on the electrode surface can be reduced to Mn^3+^, and at the same time, adsorbed analyte molecules inject electrons into Mn^4+^, completing charge compensation. This process can be quantitatively characterized by Mott–Schottky analysis and electrochemical impedance spectroscopy (EIS): in solutions containing the target analyte, the R_ct_ of the MnO_2_-modified electrode is usually significantly reduced, indicating that the analyte participates in and promotes interfacial electron transfer. Recent density functional theory (DFT) calculations further indicate that surface hydroxyl groups (-OH) and oxygen vacancies (V_0_) can effectively modulate the local work function of MnO_2_. Oxygen vacancies introduce defect energy levels into the forbidden band, reducing its band gap from approximately 2.5 eV (without defects) to 1.2–1.8 eV, thereby enabling the oxidation or reduction of analytes at lower overpotentials. Additionally, the high specific surface area and crystal facet exposure effects brought by nanostructures are also crucial: for example, the (001) facet of *δ*-MnO_2_ nanosheets possesses abundant edge sites, where the Mn atoms are coordination-unsaturated and more prone to direct interaction with electron donor analytes.

The electrocatalytic activity of MnO_2_ can be decomposed into two parts: “intrinsic activity” and “structure-induced activity”. The intrinsic activity originates from the mixed valence states (+3 and +4) of Mn. The presence of Mn^3+^ endows MnO_2_ with activities similar to those of peroxidase and superoxide dismutase. For the sensing of H_2_O_2_, the widely accepted mechanism is as follows: adsorption (H_2_O_2_ coordinates with surface Mn^4+^), electron transfer (H_2_O_2_ loses two electrons to generate O_2_, and these two electrons are transferred to the electrode through the Mn^4+^/Mn^3+^ cycle), and regeneration (Mn^3+^ is re-oxidized to Mn^4+^ by the anodic potential). The rate-determining step of this process is typically the first electron transfer. Catalytic activity can be quantified by the Tafel slope and turnover frequency (TOF). The Tafel slope of *δ*-MnO_2_ for H_2_O_2_ oxidation is often as low as 40–60 mV/dec, indicating its excellent catalytic performance at low overpotentials. The structure-induced activity originates from the edge sites, step sites, and defect centers brought about by the nano-morphology. For example, MnO_2_ nanoflowers assembled from ultrathin nanosheets expose a large number of edge oxygen vacancies and exhibit an extremely high current response to the oxidation of ascorbic acid (AA). MnO_2_ nanorods, growing along the [001] direction, primarily expose (110) and (100) surfaces, exhibiting stronger adsorption and catalytic leaching capabilities for heavy metal ions such as Pb^2+^. Mesoporous MnO_2_ possesses 3D interconnected pores, which not only provide channels for rapid diffusion of electrolyte ions but also allow a large number of dangling bonds on the pore walls to directly participate in the pre-concentration and electrocatalysis of analytes.

### 4.2. Electrochemiluminescence Sensor

An EL sensor conducts target identification utilizing an optical signal generated from electrochemical reaction-excited high-energy electron transfer of luminescent material, highlighted by high sensitivity, a broad linear range, a high signal-to-noise ratio and simple instrumentation, finding extensive applications in environmental monitoring, bioanalysis and clinical diagnostics [60]. Unlike its role in electrochemical sensors, the advantage of applying the MnO_2_ nanostructure to an EL sensor primarily lies in its efficient catalysis and the regulation of the EL process. Most EL materials generate light signals by forming active intermediates through a series of electron transfer reactions with a co-reactant. Due to its excellent electrochemical reactivity and extremely high specific surface area, MnO_2_ can serve as an efficient co-reaction promoter to accelerate the redox of co-reactants significantly. This enables the generation of more luminescent active intermediates under higher rates and lower applied voltages, thereby significantly enhancing the signal intensity and sensitivity of EL sensing. Additionally, the vast specific surface area of MnO_2_ nanostructures enables efficient loading of substantial amounts of electrochemiluminescence materials, facilitating the construction of integrated electrochemiluminescence sensors, such as sandwich-type sensors and solid-phase sensors. However, pure MnO_2_ exhibits poor conductivity and limited functionality. An electrochemiluminescence sensing platform with superior performance can be constructed by incorporating secondary functional materials, such as noble metals and conductive materials, into pure MnO_2_. The synergistic effect between highly conductive materials and MnO_2_ creates a “high-speed channel” for electron transport, thereby enhancing the signal intensity and response speed of electrochemical luminescence sensors. Precious metals can not only serve as active electrochemical-luminescent materials but also generate synergistic catalytic effects with MnO_2_, thereby enabling the construction of self-amplifying or dual-signal electrochemical luminescence sensors.

### 4.3. Photoelectrochemical Sensor

The detection principle of a PEC sensor is as follows: The separation between the charge and hole occurs on the surface of a photoactive material induced by light excitation. The electron on the surface of the photoactive material transfers from the valence band to the conduction band to generate an electron–hole pair. The formation of an electron–hole pair promotes a redox reaction on the electrode surface, subsequently generating a measurable current in the external circuit. The PEC signal is related to analyte concentration when the analyte interacts with the substrate. A PEC sensor typically exhibits multiple advantages, including high response rates, low detection limits, negligible background signals, low energy consumption and simple instrumentation. Consequently, PEC sensors have found extensive applications in food testing, bioanalysis, clinical diagnostics and environmental protection [61]. MnO_2_ serves as a direct electrocatalyst in electrochemical sensors, while it primarily synergizes with optically active materials in PEC sensors. MnO_2_ polymorphs possessing *n*-type semiconductor properties can better match numerous optically active *n*-type semiconductors. The photo-generated electron rapidly transfers from MnO_2_ to the semiconductor, while the hole transfers from the semiconductor to MnO_2_ when a “MnO_2_@*n*-type semiconductor” composite is exposed to illumination, which significantly suppresses the recombination of the electron–hole pair, thereby enabling the generation of stronger and more stable photocurrent signals. Additionally, the photoelectrical response efficiency of MnO_2_ can be further enhanced by combining with other photoactive materials or conductive materials. Therefore, the MnO_2_ nanostructure and its composites offer significant advantages for their application in novel PEC sensors.

### 4.4. Colorimetric Sensor

Colorimetric sensors have merits such as high sensitivity, high selectivity, high detection efficiency, simple fabrication and ease of visual identification, making them widely used for detecting toxic pollutants, like metal ions, organic dye and pesticides [62]. In enzyme-mediated and enzyme-mimetic colorimetric sensors, analyte detection is carried out based on color changes induced by the redox of 3,3,5,5-tetramethylbenzidine catalyzed by a natural or bioinspired enzyme. The advantages of utilizing MnO_2_ in colorimetric sensors include its unique enzyme-like catalytic activity and its inherent property as a color indicator. MnO_2_ possesses intrinsic oxidase-like activity, which is capable of oxidizing colorless substrates into colored ones without H_2_O_2_. Compared to a natural enzyme, a MnO_2_ bioinspired enzyme offers lower cost, higher stability and stronger colorimetric signal intensity. Furthermore, a nano-MnO_2_ solution can function as a color indicator, making it simple and convenient for colorimetric sensing. Specifically, dark Mn^4+^ is reduced to transparent Mn^2+^ upon the addition of a reducing analyte. Notably, when MnO_2_ is combined with a noble metal nanoparticle, a synergistic catalytic effect between them significantly enhances the enzyme-like catalytic activity, resulting in a faster color reaction and deeper color. Therefore, it is worth exploring the application of the MnO_2_ nanostructure and its composites in novel colorimetric sensors.

### 4.5. Fluorescent Sensor

As a type of colorimetric sensor, a fluorescence sensor detects the target by monitoring changes in fluorescence signals in fluorophore after reacting with analytes [63]. The MnO_2_ nanostructure exhibits high fluorescence quenching efficiency, which regulates the “on” and “off” of the fluorescence signal through both the Förster resonance energy transfer (FRET) and the inner filter effect (IFE). FRET only occurs over a very short distance. Specifically, the fluorescent molecule will transfer energy to nearby MnO_2_ nanomaterials through dipole–dipole interaction instead of emitting photons directly, resulting in fluorescence quenching. On the other hand, IFE can occur over a long distance. Particularly, when fluorescent molecules and MnO_2_ nanomaterials coexist, the strong absorption spectrum of MnO_2_ overlaps with the excitation or emission spectrum of fluorescent molecules, resulting in a decrease in fluorescence signals. Fluorescent sensors have been widely adopted in environmental monitoring and medical diagnostics due to their high sensitivity, rapid response and non-destructive analysis. Notably, the 2D planar structure of a MnO_2_ nanosheet maximizes face-to-face contact and interaction between MnO_2_ and fluorophore molecules, significantly enhancing fluorescence quenching efficiency and sensing sensitivity. Furthermore, integrating the MnO_2_ nanostructure with a Au nanorod or mesoporous SiO_2_ can combine the strength of multiple materials to achieve more complex sensing. Consequently, it is worth exploring the application of MnO_2_ and its composites in novel fluorescent sensors.

### 4.6. Photothermal Sensor

In a photothermal sensor, target analysis is carried out through detecting temperature changes originating from a photothermal conversion substrate which absorbs light energy and converts it into thermal energy. The low cost, simple operation, easy data readout and portability are main highlights of photothermal sensing, making it widely applicable in environmental monitoring, biomedical research and industrial control [17]. The detection mechanism of a photothermal sensor refers to the photothermal effect. The photothermal conversion substrate generates heat *via* electron transition or vibration under an external light source, while the produced heat can be altered when the photothermal material interacts with the target substance, enabling concentration sensing of analytes. The MnO_2_ nanostructure exhibits outstanding photothermal conversion efficiency. Upon absorbing infrared light, MnO_2_ converts light energy into heat through a non-radiative relaxation process, causing an increase in ambient temperature. MnO_2_-based photothermal sensors can be divided into “signal-off” sensing based on chemical reduction and “signal-on” sensing in which MnO_2_ acts as a carrier or quencher. Specifically, in “signal-off” sensing, in the presence of a reducible target, MnO_2_ with high infrared absorption activity is reduced to Mn^2+^ with extremely weak infrared absorption, resulting in the weakening or disappearance of the substrate’s photothermal effect and a decrease in environmental temperature. In “signal-on” sensing, other photothermal nanomaterials can be loaded on the MnO_2_ nanosheet. Before adding the target substance, MnO_2_ will quench or encapsulate the photothermal effect of other materials. Upon adding the target substance, MnO_2_ is decomposed, and then the photothermal material is released, and the photothermal effect is restored. In photothermal sensing, MnO_2_ is typically combined with other highly photothermally active materials, including precious metals, copper sulfide and polydopamine. The synergistic effect between MnO_2_ and photothermally active materials yields enhanced photothermal effects and stronger temperature signals, thereby improving the sensitivity and signal-to-noise ratio of photothermal sensing. Therefore, applying MnO_2_ and its composites in novel photothermal sensors holds great significance.

### 4.7. Quartz Microbalance Sensor

QCM sensing is based on the piezoelectric effect, analyzing the target substance by measuring the impact of the adsorbed mass on the resonant frequency of a quartz crystal within an alternating electric field. Characterized by ultra-high sensitivity and real-time dynamic monitoring, a quartz crystal microbalance sensor finds extensive applications in biosensing and environmental monitoring [18]. MnO_2_ nanostructures possess extremely high specific surface areas and abundant pores. They can not only efficiently adsorb and enrich target analytes through physical adsorption or chemical interactions but also significantly “amplify” the mass of the target molecule into mass change across the entire nanomaterial layer. This effect substantially enhances the frequency response signal of QCM sensing, thereby improving detection sensitivity. Notably, pure MnO_2_ suffers from poor conductivity and structural instability under specific conditions, and the durability and sensitivity of QCM sensors can be enhanced by combining pure MnO_2_ with conductive polymers. Consequently, applying nano-MnO_2_ and its composites to novel QCM sensors holds considerable significance.

## 5. Manganese Dioxide Nanostructure-Based Novel Sensing in Environmental Monitoring, Food-Safety Monitoring and Biomedical Monitoring

MnO_2_ nanostructures, encompassing 0D, 1D, 2D and 3D nanostructures, have been extensively applied in environmental monitoring, including air, water and soil pollutants, as well as food-safety monitoring and biomedical monitoring. Novel sensing technologies include electrochemistry, electrochemiluminescence, photoelectron chemistry, colorimetry, fluorescence, photothermal techniques and QCM. In this section, based on different application fields and multiple sensing technologies in a specific field, the application of MnO_2_ nanostructure-based novel sensing in various fields over the past five years are summarized, particularly focusing on sensing performance and corresponding mechanisms, and the improvement of sensing performance by MnO_2_ microstructure.

### 5.1. Environmental Monitoring

#### 5.1.1. Air Pollutant Monitoring

Gas sensing is primarily carried out based on electrochemical technology, detecting the target gas by monitoring conductivity changes in sensing substrates under different gas environments. The process involves multiple theories, including the chemisorption oxygen model, grain boundary barrier model, bulk resistance model and space charge layer model. The core principle of gas sensing lies in the interaction between the gas and substrate surface. 0D, 1D, 2D and 3D MnO_2_ can be utilized for air pollutant monitoring. Common air pollutants include NH_3_, NO_2_, HCHO, CH_3_COOH, CH_3_CHO, hexanal, triethylamine and allyl mercaptan.

The application of MnO_2_ nanostructures and their composites in air pollutant monitoring over the past five years have been summarized in Table 1, particularly including the morphology of MnO_2_, the composition of the substrate and the analyte, the limit of detection (LOD), the response and response time of the sensor, and the corresponding reference.

##### MnO_2_ Nanostructure-Based Non-Heterostructure Composite

In order to enhance sensing performance and broaden application scenarios, compositing pure MnO_2_ with other materials or functional modifications of MnO_2_ are essential.

**Figure 5:**
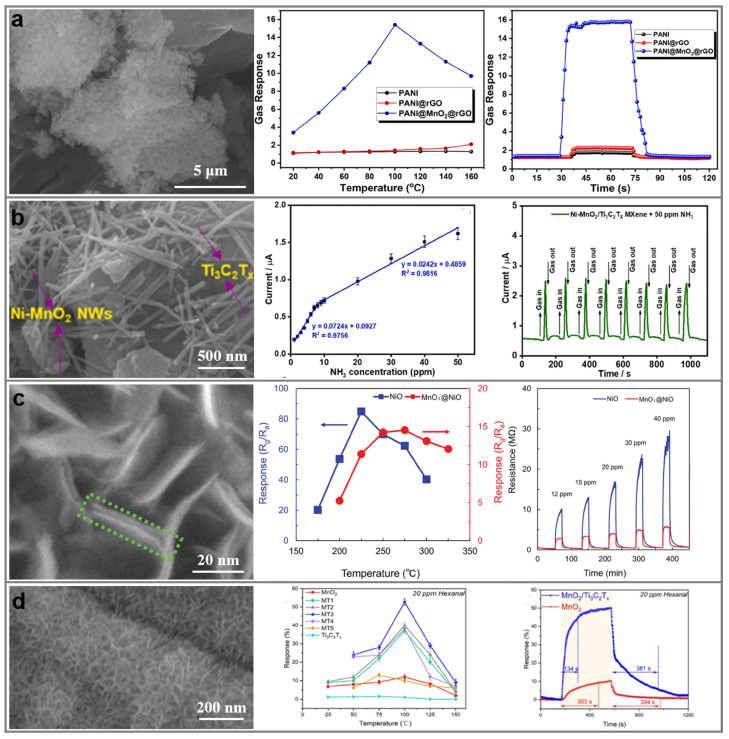
MnO_2_ nanostructure-based novel sensing for air pollutant monitoring. (**a**) PANI@MnO_2_@rGO ternary nanocrystal for NH_3_ detection: SEM image of ternary nanocrystal, response rate of ternary nanocrystal for 50 ppm of NH_3_ gas at different operating temperatures, and response–recovery time curve of ternary nanocrystal for 50 ppm of NH_3_ at the optimized temperature of 100 °C; (**b**) Ni-MnO_2_/Ti_3_C_2_T_x_·MXene for NH_3_ detection: SEM image of composite material, calibration curve of the composite for low and high NH_3_ concentration at +1.1 V, and response–recovery time curve of the composite material for NH_3_ at optimized temperature; (**c**) MnO_2_@NiO nanosheet for allyl mercaptan detection: SEM image of MnO_2_@NiO nanosheet, response rate of MnO_2_@NiO nanosheet-based gas sensor for 30 ppm of allyl mercaptan at different operating temperatures, and gas sensing dynamic curve of MnO_2_@NiO nanosheet exposed to varying allyl mercaptan concentrations at optimal operating temperatures; (**d**) MnO_2_/Ti_3_C_2_T_x_ composite for detecting lung cancer biomarker hexanal: SEM image of the composite, response value of composite-based gas sensor for 20 ppm of hexanal at different operating temperatures (25–150 °C), and response–recovery time curve of the composite for 20 ppm of hexanal at optimized temperature.

Zeng et al. reported a simple process for preparing a sensing composite *via* physical blending of PANI-loaded reduced graphene oxide (rGO) and MnO_2_ nanoparticles. Specifically, firstly, a MnO_2_ nanoparticle and rGO were prepared *via* acid-assisted hydrothermal reduction pyrolysis and the Hummer-hydrazine reduction method, respectively. Subsequently, the ternary nanocomposite PANI@MnO_2_@rGO was assembled through in situ oxidation polymerization-solution self-assembly [64] (Figure 5a). For PANI alone and PANI@MnO_2_, the measured gas response-recovery profiles did not exhibit significant changes upon exposure to NH_3_ gas. A PANI@MnO_2_@rGO-based sensor exhibits remarkable selectivity toward NH_3_, demonstrating a response value of 15.56 at 100 °C for 50 ppm NH_3_, with response and recovery times of 6 and 10 s, respectively. The sensing mechanism of PANI@MnO_2_@rGO involves an oxygen-containing anion on the surface: The O_2_ is absorbed on the surface of the ternary composite. The adsorbed O_2_ molecule undergoes dissociation by extracting electrons from the conduction band of the *n*-type MnO_2_ nanoparticle, generating multiple oxygen-containing anion species. These oxygen-containing anion species react with NH_3_, thereby reducing the resistance of the substrate and enhancing sensing performance. Furthermore, the incorporation of MnO_2_ and rGO enhances the gas adsorption site, promotes charge transfer and increases the overall material conductivity, collectively elevating sensitivity, ensuring effective responsibility for changes in NH_3_ concentration and retaining outstanding stability.

In addition to physical blending with other substrates, heteroatom doping is also an effective method to enhance the sensing performance of pure MnO_2_. Benefiting from the synergistic effect of chemical and electronic sensitization from heteroatoms, the surface activity of substrate materials can be significantly improved, thereby promoting the catalytic decomposition of gas molecules. Mari et al. synthesized a composite material consisting of a Ni-doped MnO_2_ nanowire (Ni-MnO_2_ NWs) and Ti_3_C_2_T_x_·MXene (Ni-MnO_2_/Ti_3_C_2_T_x_·MXene) using a one-step hydrothermal method and self-assembly technique applied in NH_3_ detection based on the electrochemical method under room temperature [65] (Figure 5b). There exist significant structural advantages in Ni-MnO_2_/Ti_3_C_2_T_x_·MXene: The 3D hierarchical architecture is formed through loading of Ni-MnO_2_ NWs onto a multilayer Ti_3_C_2_T_x_·MXene surface uniformly with the nanowire tightly integrating into the MXene layer. Additionally, more oxygen vacancies are introduced, and a Mn^3+^/Mn^4+^ mixed oxidation state is formed by Ni doping. Moreover, Brunauer–Emmett–Teller (BET) analysis reveals that the composite material possesses a specific surface area of 85.5 m^2^/g, which is 26 times higher than that of pure MXene. X-ray photoelectron spectroscopy (XPS) analysis was performed to assess the elemental composition and electronic properties of MnO_2_, Ni-MnO_2_, Ti_3_C_2_T_x_·MXene, MnO_2_/Ti_3_C_2_T_x_·MXene, and Ni-MnO_2_/Ti_3_C_2_T_x_·MXene electrode materials. The multiple deconvolutions observed in each material indicate several contributions to the MnO_2_ spectra, likely due to the presence of Mn^4+^/Mn^3+^ oxidation states. Ni-MnO_2_/Ti_3_C_2_T_x_·MXene suggests a lower Mn oxidation state than in *α*-MnO_2_, likely due to interactions with Ni in Ni-MnO_2_/Ti_3_C_2_T_x_·MXene, creating oxygen vacancies that improve structural stability and electrochemical reversibility. These structural advantages of Ni-MnO_2_/Ti_3_C_2_T_x_·MXene endow it with outstanding performance toward NH_3_ sensing under optimized conditions, with a sensitivity of 0.072 μA/ppm, a detection limit as low as 0.23 ppm, a response/recovery time of only 45 s/79 s (at 20 ppm NH_3_), bilinear response across a NH_3_ concentration of 1–50 ppm, along with outstanding selectivity, repeatability and long-term stability. Compared to a bare SPCE, the MnO_2_, NiMnO_2_, Ti_3_C_2_T_x_·MXene, Ni/Ti_3_C_2_T_x_·MXene, MnO_2_/Ti_3_C_2_T_x_·MXene, Ni-MnO_2_/Ti_3_C_2_T_x_·MXene, and Ni-MnO_2_/Ti_3_C_2_T_x_·MXene-modifed electrodes exhibit the highest chronoamperometric current response. The response/recovery times for the Ni-MnO_2_/Ti_3_C_2_T_x_·MXene sensor were 45 s/79 s, respectively, which are 1.18/1.50, 1.68/1.24,1.40/1.09, 1.22/1.31, 1.46/1.25, and 1.06/1.13 times shorter than those of the bare electrode and the MnO_2_, Ni-MnO_2_, Ti_3_C_2_T_x_·MXene, Ni/Ti_3_C_2_T_x_·MXene, and MnO_2_/Ti_3_C_2_T_x_·MXene ones, respectively. In other words, it exhibited the shortest response/recovery times. It is noteworthy that Ni-MnO_2_/Ti_3_C_2_T_x_·MXene has been successfully applied to fish meat freshness monitoring. The sensing primarily relies on releasing electrons that originated from a three-electron oxidation reaction between NH_3_ and [TFSI]^−^ in ionic liquid electrolytes. Ni-MnO_2_/Ti_3_C_2_T_x_·MXene enhances the signal intensity through a triple synergistic effect, in which Ni acts as an electrocatalyst to promote NH_3_ oxidation, MnO_2_ NWs provide a reaction site *via* high adsorption capacity and oxygen vacancy, and MXene accelerates electron transport due to its high conductivity.

By doping MnO_2_ or physically blending it with other sensor substrates, the synergistically coupling advantage of different active materials can effectively enhance the sensitivity, selectivity, repeatability and long-term stability of gas sensing.

##### MnO_2_ Nanostructure-Based Heterojunction Composite

For MnO_2_ sensing materials prepared *via* physical blending without chemical bonding interaction, issues including substrate separation and degraded sensing performance after prolonged operation often exist. Conversely, sensing stability can be enhanced by constructing a heterostructure with robust chemical bonding interaction. It is well-established that forming a heterostructure between two semiconductors is effective for improving gas-sensing performance. Strong interaction at the heterointerface significantly influences oxygen adsorption, catalytic activity and charge transfer, thereby further enhancing sensor response.

Li et al. synthesized a NiO nanosheet and MnO_2_@NiO nanosheet *via* a sol-gel and etching-recrystallization method for detecting allyl mercaptan (AM), a marker gas of psychological stress release, thereby assessing stress levels [1] (Figure 5c). Specifically, the particular structure generated from the attachment of the *p-p* heterojunction consists of MnO_2_ which vertically aligns with a porous NiO nanosheet that contributes to superior sensing performance. Fundamentally, the gas sensing of the MnO_2_@NiO nanosheet operates primarily through the release of electrons when the AM gas reacted with adsorbed oxygen on its surface, leading to a decrease in resistance. The response time and recovery time are closely related to the detection speed of the sensor, and they are two important parameters for the evaluation of gas sensing performance. The values of response time and recovery time of the NiO and MnO_2_@NiO sensor were respectively 480 and 15 s, and 115 and 25 s at 40 ppm of AM vapor. Compared to a NiO nanosheet-based sensor, this composite-based sensor exhibits a faster response time for 40 ppm of AM gas. Additionally, the *p-p* heterojunction in MnO_2_@NiO accelerates the response rate *via* interfacial charge transfer, and the porous structure and high oxygen adsorption capacity of NiO contribute to a higher response value.

Additionally, direct loading MnO_2_ onto the substrate *via* chemical bonding interaction represents another effective modification strategy. This approach not only enables efficient dispersion of MnO_2_ on the substrate to prevent agglomeration but also enhances the selectivity and responsiveness of MnO_2_ through heterojunction regulation. Yao et al. synthesized a MnO_2_/Ti_3_C_2_T_x_ composite *via* a one-step hydrothermal method for the detection of hexanal gas, a lung cancer biomarker, with high sensitivity and selectivity [57] (Figure 5d). In particular, the composite was constructed by wrapping a MnO_2_ nanosheet tightly on Ti_3_C_2_T_x_·MXene, thereby forming a *p-p* heterojunction structure with abundant oxygen vacancies and high catalytic activity. Compared to pure MnO_2_ nanospheres, the composite demonstrated a higher response value, namely 52% response, for 20 ppm of hexanal at 100 °C, with response and recovery times of 134 s and 381 s respectively, as well as an excellent linear range, resistance to humidity interference and long-term stability. The outstanding sensing performance of MnO_2_/Ti_3_C_2_T_x_ primarily stems from the carrier regulation at the *p-p* heterojunction: O_2_ molecules adsorbing in air form a hole accumulation layer (HAL), which thins upon hexanal contact due to electron–hole recombination, causing a significant resistance increase. Concurrently, high catalytic selectively of MnO_2_ promotes hexanal oxidation. In summary, the heterojunction interface accelerates carrier separation and transport, and the synergistic interaction between the conductive network of MXene and the catalytic active site of MnO_2_ coupled with the oxygen vacancy and defect site provide additional gas-reactive centers, thereby combining high responsivity and unique selectivity under low temperatures. A more detailed explanation of how these vacancies are intentionally characterized is necessary. The XRD diffraction peak corresponding to the *δ*-MnO_2_ of MnO_2_/Ti_3_C_2_T_x_ became very weak because of the growth of non-oriented layered MnO_2_ nanosheets on the surface of MXene, or the existence of a large number of oxygen vacancies in MnO_2_ after compounding with MXene. The low-binding energy OII peak at 531.4 eV in the XPS spectrum corresponds to oxygen vacancies, adsorbed oxygen, or M (Mn, Ti)-OH bonds. It is worth noting that the Mn^3+^/Mn^4+^ strength of MnO_2_/Ti_3_C_2_T_x_ in the Mn 2p XPS spectra of MnO_2_/Ti_3_C_2_T was much greater than that of MnO_2_, indicating that the amount of Mn^3+^ ions in MnO_2_ nanosheets on the surface of MnO_2_/Ti_3_C_2_T_x_ greatly increased. The rise in the Mn^3+^ amount indicates a high electron cloud density around Mn sites, leading to more oxygen vacancies.

#### 5.1.2. Pollutant Monitoring in Water Quality and Soil

MnO_2_ nanostructure-based sensors utilizing electrochemical techniques, PEC techniques, colorimetric techniques, fluorescence techniques, photothermal techniques, QCM techniques, microfluidic techniques and solid-phase extraction adsorption techniques can be employed for monitoring pollutants in water and soil. Common water and soil pollutants include heavy metal ions, such as Cr, As, Se, Sr, Hg, and Pb; N_2_H_4_·H_2_O; organophosphorus pesticides; antibiotic drugs, such as tetracycline and chloramphenicol; sulfonamide drugs, such as sulfadiazine; phenolic compounds, such as catechol, resorcinol and tetrabromobisphenol A; diethyl phthalate; malachite green; as well as rifampicin.

Applications of MnO_2_ nanostructures in monitoring water and soil pollutants over the past five years have been summarized in Table 2, mainly including the sensing mechanism, the morphology of MnO_2_, the composition of the substrate and analyte, the linear detection range and the minimum detection limit of the analyte, along with the corresponding reference.

##### MnO_2_ Nanostructure-Based Electrochemical Sensing

For detecting dangerous pathogens, traditional methods such as biological culture colony counting are time-consuming and labor-intensive. Electrochemical sensing has garnered significant attention in pathogen detection due to its simplicity, cost-effectiveness, sensitivity, ease of miniaturization and resistance to interference.

**Figure 6:**
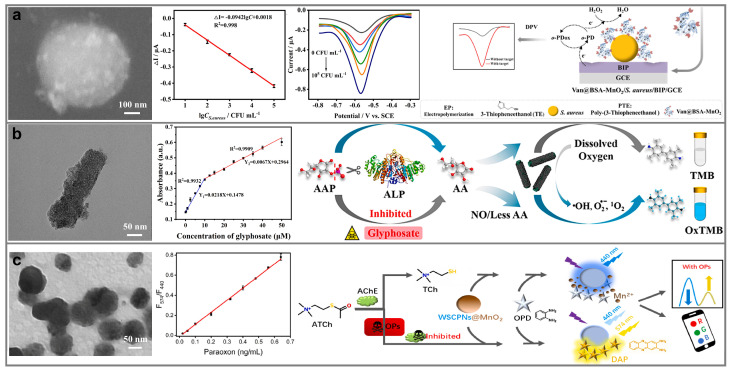
MnO_2_ nanostructure-based novel sensing for pollutant monitoring in water quality and soil. (**a**) Electrochemical technology-based Staphylococcus aureus detection utilizing Van@BSA-MnO_2_ nanoparticle as the probe: SEM image of Van@BSA-MnO_2_, calibration curve for Staphylococcus aureus with different concentrations, differential pulse voltammetry response rates for Staphylococcus aureus with various concentrations, and detection mechanism for Staphylococcus aureus based on electrochemical technology utilizing Van@BSA-MnO_2_ as the probe; (**b**) detection of OPs using PANI-MnO_2_ nanorod-based colorimetric sensing platform: TEM image of PANI-MnO_2_, calibration curve for glyphosate detection with different concentrations of 0–50 μM, and detecting mechanism of OPs using PANI-MnO_2_ nanorod-based colorimetric technology; (**c**) OP detection based on fluorescence technology utilizing WSCPNs@MnO_2_ nanoparticle as the probe: TEM image of WSCPNs@MnO_2_, linear calibration curve for oxygen and phosphorus detection using WSCPNs@MnO_2_-based fluorescence technology with a ratio of *n* = 3, and detecting mechanism for OPs utilizing WSCPNs@MnO_2_-based fluorescence technology.

Ma et al. constructed a multifunctional and low-cost detection platform for detecting Staphylococcus aureus based on a composite material [27] (Figure 6a). Vancomycin (Van) was first coupled to bovine serum albumin (BSA) *via* a bacterial imprinting polymer (BIP), which was then loaded onto MnO_2_, and the Van@BSA-MnO_2_ composite was synthesized. Upon binding with captured Staphylococcus aureus, the reaction between the substrate and target was catalyzed by the Van@BSA-MnO_2_ complex, generating an electrical signal that is proportional to the concentration of the bacterial target. The Van@BSA-MnO_2_-based sensor exhibits remarkable sensitivity, being capable of detecting a single bacterial cell in phosphate-buffered saline. The sensor exhibits outstanding selectivity, particularly in distinguishing target Staphylococcus aureus from interfering bacteria of the same genus at a concentration of up to 100 times higher. Additionally, to assess the applicability of the sensor in intricate food matrices, experiments were performed using milk as a representative example. To reduce the matrix’s interference, milk was diluted 10-fold and spiked with varying concentrations of S. aureus. The recovered S. aureus cells were then analyzed. Of particular significance is the assay’s sensitivity, as evidenced in its ability to detect S. aureus concentrations as low as 10 CFU/mL.

##### MnO_2_ Nanostructure-Based Colorimetric Sensing

Due to its advantages of rapid response, direct visual observation and ease of operation, colorimetry is commonly regarded as a widely applicable environmental detection method.

It is crucial for designing high-performance colorimetric sensors through preparing advanced functional nanostructures with high specificity and catalytic performance, such as synergistically coupling other sensing substrates with MnO_2_ to further optimize catalytic activity. Yang et al. synthesized a composite material of PANI loaded with MnO_2_ nanoparticles (PANI-MnO_2_) *via* one-step self-assembly redox polymerization under room temperature to construct a smartphone-based colorimetric sensing platform for detecting organophosphorus pesticides (OPs) [72] (Figure 6b). This composite consists of rod-like PANI as the substrate and MnO_2_ nanoparticles uniformly distributed on its surface, forming a core–shell heterojunction with a mesoporous structure. The oxidase activity of PANI-MnO_2_ is inhibited by catalyzing the conversion of ascorbic acid-2-phosphate (AAP) to ascorbic acid (AA) *via* alkaline phosphatase (ALP). Under the same conditions, the relative catalytic activity is in the order of PANI-MnO_2_ > MnO_2_ nanosheets > MnO_2_. The redox peak currents of PANI-MnO_2_/GCE were higher than MnO_2_ nanosheets/GCE and MnO_2_/GCE, demonstrating the better conductivity and cycling stability of PANI-MnO_2_/GCE. On the other hand, the oxidation capacity for 3,3′,5,5′-tetramethylbenzidine (TMB) of PANI-MnO_2_ can be restored through adding OPs, which inhibits the activity of ALP, thereby achieving a linear detection range of 0.50–50 μM with a detection limit as low as 0.39 μM. This unique core–shell architecture enables the material to exhibit a 28.7% higher catalytic efficiency than pure MnO_2_ at pH 4.0. Furthermore, to explore the practical usability of the proposed colorimetric method, we determined the glyphosate concentration in tap water, lake water, soil, pear, soybean and cucumber. Among them, OPs were detected in the soybean sample but were not detected in the other samples. The recovery rate was in the range of 98.4–108.6%, and the relative standard deviation (RSD) was within the range of 0.6–3.7%. All these results indicate the feasibility of the proposed method for detecting glyphosate in practical samples. The underlying sensing mechanism involves color development through oxidizing TMB by reactive O species generated from dissolved O_2_ oxidizing catalyzed by MnO_2_. The introduction of PANI not only enriches TMB molecules *via* electrostatic adsorption but also accelerates electron transfer through an extended *π*-conjugated system. Concurrently, the specific surface area is increased, and the active site is exposed due to the specific mesoporous structure of PANI-MnO_2_.

##### MnO_2_ Nanostructure-Based Fluorescent Sensing

There exist a variety of advantages of fluorescence sensing, such as high sensitivity, broad applicability, controllable cost and rapid analysis. Furthermore, ratio fluorescence detection technology, which measures the ratio of fluorescence with two different wavelengths, can effectively mitigate the impact of the fluorescence background and environmental factor, further enhancing sensing interference resistance.

Li et al. synthesized a water-soluble fluorescent conjugated polymer nanoparticle (WSCPNs)@MnO_2_ (WSCPNs@MnO_2_) probe using WSCPNs *via* nanoprecipitation for the highly sensitive and visual fluorescence-based detection of OPs [70] (Figure 6c). This sensing material consists of poly(9,9-di-n-octylfluorene-2,7-diyl) (PFO) as the fluorescent core and polystyrene-maleic anhydride (PSMA) as the amphiphilic shell. MnO_2_ was anchored onto the surface of WSCPNs *via* in situ synthesis, forming a spherical structure. The fluorescence emission peak of WSCPNs highly overlaps with the absorption spectrum of MnO_2_, enabling efficient FRET with a fluorescence quenching efficiency as high as 99%. In the process of sensing, the conversion of acetylthiocholine (ATCh) to thiocholine (TCh) is first catalyzed by acetylcholinesterase (AChE), and then the MnO_2_ is reduced to Mn^2+^ catalyzed by TCh, restoring the blue fluorescence of WSCPNs. While in the presence of OPs, the activity of AChE is inhibited, and the oxidation of o-phenylenediamine (OPD) to form 2,3-diaminophenazine (DAP) catalyzed by residual MnO_2_ contributes to the emitting of yellow fluorescence. Quantitative analysis was achieved by monitoring the fluorescence intensity ratio at 440 nm and 574 nm, with a detection limit as low as 0.0139 ng/mL. And the detection limit of smartphone RGB analysis visualization is 0.025 ng/mL. The in situ synthesized compact structure not only enhances FRET efficiency but also improves the photostability and interference resistance of sensing materials. The practicality of this method was evaluated by spiking paraoxon into leaves and soil. The samples, spiked with paraoxon standards at concentrations of 100 ng/mL, 200 ng/mL, and 300 ng/mL, were measured using the ratiometric fluorescence mode, the fluorescence visualization mode, and the gas chromatography–mass spectrometry (GC-MS) method as the standard. The fluorescence visualization revealed that the recovery values ranged from 96.7 to 99.9% for tree leaves and 97.6–101.4% for soil. Using ratiometric fluorescence, the recovery values were in the range of 97.6–100.7% for tree leaves and 95.7–103.1% for soil. These results confirm the accuracy and reliability of the ratiometric fluorescence probe for detecting paraoxon in real samples. This compact structure also minimizes non-specific adsorption through uniform coverage, significantly improving the probe’s sensitivity and stability. The paper provides a low-cost, highly sensitive dual-mode sensing platform for environmental monitoring.

### 5.2. Food-Safety Monitoring

Common food contaminants include nitrite, dopamine, antioxidants, Salmonella enterica, aflatoxin, ochratoxin A, trithion, dithiocarbamate and dichlorvo. MnO_2_ nanostructure-based novel sensing technologies for food-safety monitoring include electrochemical methods, colorimetric methods, fluorescence methods, photothermal methods, lateral flow immunoassays and microfluidic sensing.

Applications of MnO_2_ nanostructure-based novel sensing technology in food-safety monitoring over the past five years have been summarized in Table 3, including the sensing mechanism, the morphology of MnO_2_, the chemical composition of the substrate and analyte, the linear detection range and the minimum detection limit of the analyte, and the corresponding reference.

#### 5.2.1. MnO_2_ Nanostructure-Based Electrochemical Sensing

The MnO_2_ nanostructure and its composites offer exceptionally prominent advantages for their application in novel electrochemical sensor-based food-safety monitoring.

**Figure 7:**
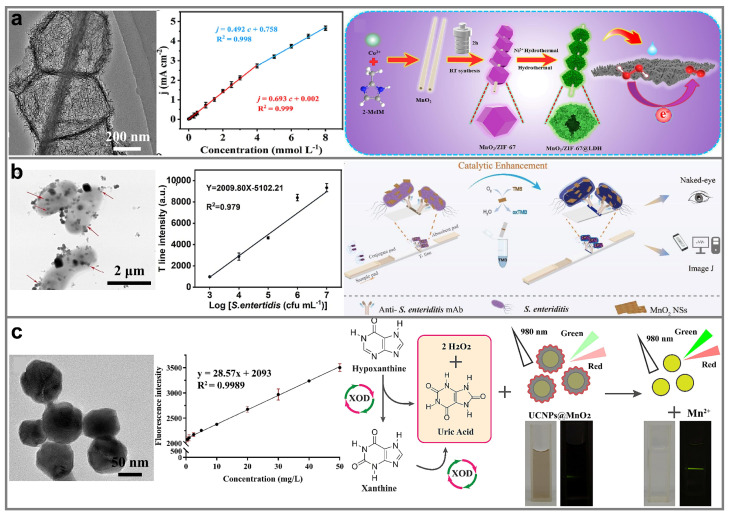
MnO_2_ nanostructure-based novel sensing for food-safety monitoring. (**a**) H_2_O_2_ detection utilizing MnO_2_/ZIF-67@LDH-based electrochemical technology: TEM image of MnO_2_/ZIF-67@LDH, calibration curve of catalytic oxidation current versus H_2_O_2_ concentration for MnO_2_/ZIF-67@LDH, and detection mechanism for H_2_O_2_ utilizing MnO_2_/ZIF-67@LDH-based electrochemical technology; (**b**) detection of Salmonella enterica based on amplified colorimetric signals utilizing MnO_2_ NSs as the substrate: TEM image of MnO_2_ NSs, standard curve fitting of T-line signal intensity versus Salmonella enterica concentration after catalyzing, and detection mechanism for Salmonella enterica; (**c**) the detection of hypoxanthine based on fluorescence turn-on technology utilizing MnO_2_@UCNPs as the substrate: TEM image of MnO_2_@UCNPs, linear fitting of upconversion fluorescence intensity of green peak in UCNPs@MnO_2_@XOD nanocomposite at different hypoxanthine concentrations of 0.5, 1, 2.5, 5, 10, 20, 30, 40, and 50 mg/L, and detecting mechanism for hypoxanthine using MnO_2_@UCNP-based fluorescence technology.

H_2_O_2_ is commonly added to food as a disinfectant or bleaching agent during processing, but excessive H_2_O_2_ residuals will seriously affect human health. Xiong et al. synthesized a hierarchical porous gourd-like MnO_2_/ZIF-67@Ni-Co layered double hydroxide composite (MnO_2_/ZIF-67@LDH) *via* a multi-step hydrothermal strategy, serving as the substrate for electrochemistry-based H_2_O_2_ detection, with advantages in rapidity, sensitiveness and accuracy [81] (Figure 7a). The synthesis strategy for MnO_2_/ZIF-67@LDH was as follows: First, a MnO_2_ nanotube was prepared *via* a one-step hydrothermal approach based on the reduction of KMnO_4_ using concentrated HCl. Subsequently, a ZIF-67 nanopolyhedron was assembled onto the MnO_2_ nanotube through a secondary hydrothermal process to form MnO_2_/ZIF-67. Finally, Ni-Co layered double hydroxide nanocages were deposited onto MnO_2_/ZIF-67 *via* a third hydrothermal step. The formation process of the multistage structure in the MnO_2_/ZIF-67@LDH composite was as follows: the formation of a MnO_2_ hollow nanotube; the formation of a 3D nano-polyhedral structure by assembling the ZIF-67 polyhedron on the surface of the MnO_2_ nanotube; the transformation of a solid dodecahedral structure to a hollow dodecahedral structure *via* solvothermal etching of the ZIF-67 template; and the formation of a 3D hollow nanocage structure by stacking Ni-Co layered double hydroxide onto a hollow polyhedral framework of ZIF-67. The catalyst materials could be arranged in increasing order of current density as follows: ZIF-67@LDH < MnO_2_ < MnO_2_/ZIF-67@LDH. Accordingly, the MnO_2_/ZIF-67@LDH composite electrode has the highest activity. The designed electrochemical sensor for H_2_O_2_ detection exhibits two linear detection ranges, namely 1 × 10^−3^–4 mmol/L and 4–8 mmol/L. Sensitivity is as high as 693 µA·L/mmol·cm^2^, and the sensor has an extremely rapid response of t < 3 s and excellent reproducibility and stability. The hollow structure of the MnO_2_/ZIF-67@LDH composite provides multiple pathways and abundant cavities, facilitating electron transfer and H_2_O_2_ transport. Simultaneously, the hollow architecture of the composite offers sufficient residual space to accommodate volume and dimensional changes induced by multiple electrochemical reactions, ensuring the repeatability and stability of the sensor. Furthermore, the interconnected LDH nanosheet provides abundant active sites and a large specific surface area, enhancing sensing efficiency. The feasibility of using the MnO_2_/ZIF-67@LDH electrode for the detection of H_2_O_2_ was evaluated in real samples of milk and juice using the standard addition method. Under optimal conditions, 20 µL of a milk supernatant was added to a 0.1 M of a NaOH solution containing H_2_O_2_. The recoveries and the RSDs range from 95.2% to 114.2% and 1.47–3.16%, respectively. These results demonstrate the effectiveness and feasibility of the proposed sensor in the detection of H_2_O_2_ in food.

#### 5.2.2. MnO_2_ Nanostructure-Based Colorimetric Sensing

The key for applying MnO_2_ in colorimetric sensing primarily stems from its unique enzyme-like catalytic activity. Therefore, applying the MnO_2_ nanostructure and its composites in novel colorimetric sensors warrants further research.

Shigella enteritis in pathogenic bacteria is one of the primary risk factors for causing disease infection, which typically transmits to humans through the consumption of contaminated animal-derived food. Therefore, sensitive and rapid detection of Shigella enteritis is of significant importance to global public health security. Zhang et al. employed 2D MnO_2_ nanosheets (MnO_2_ NSs) with advantages of strong bacterial adsorption capacity, excellent optical signals and good catalytic activity as the substrate to achieve rapid, sensitive and accurate detection of Salmonella enterica *via* leveraging their oxidase-like catalytic activity to amplify colorimetric signals [84] (Figure 7b). The MnO_2_ NSs were synthesized using a one-step precipitation strategy based on the reduction of KMnO_4_ by 2-morpholinoethane sulfonic acid. X-ray diffraction (XRD) and TEM revealed that the synthesized *γ*-MnO_2_ exhibits a 2D sheet structure with an average size of approximately 250 nm. As there are different morphologies of MnO_2_ besides MnO_2_ NSs, MnO_2_ NWs were also synthesized as controls to demonstrate the superiority of MnO_2_ NSs. A performance comparison of these two morphologies of MnO_2_ shows that MnO_2_ NSs have a slight advantage. A colorimetric sensor based on MnO_2_ NSs demonstrated a linear range of 10^3^–10^7^ CFU/mL and a detection limit of 10^3^ CFU/mL for Shigella enteritis. MnO_2_ NSs perform the following functions simultaneously during colorimetric sensing: capturing bacteria as antibody mimics; providing strong colorimetric signals utilizing their brown color; and generating additional catalytic signals by amplifying oxidation activity of mimicking enzymes *via* immersion. The detection mechanism of MnO_2_ NS-based colorimetric sensing for Salmonella enterica is as follows: the formation of a substrate–analyte complex through absorbing MnO_2_ NSs onto the surface of the target bacteria; the formation of a brown band *via* special binding between the anti-Salmonella enteritidis monoclonal antibody and detection target when the substrate–analyte complex migrates to the T line, where the concentration of the bacterial target is positively linearly correlated with the color intensity of the T line; and the formation of a blue band that enhances detection sensitivity due to the pseudo-oxidase activity of MnO_2_ NSs, which oxidizes TMB into ox-TMB. The unique 2D sheet structure of MnO_2_ NSs facilitates adsorption of the analyte through electrostatic and hydrophobic interaction. In order to demonstrate the practical application of the developed MnO_2_ NSs, several actual samples were selected for testing, and a series of different concentrations of bacterial suspension were added to these food samples. As the concentration of the bacteria decreases, the intensity of the brown band of the T-line gradually becomes weaker, until it stops at an LOD concentration of 104, 104, 104 and 105 cfu·mL^−1^ for orange juice, drinking water, milk, and beef, respectively. After TMB catalytic coloration, the LOD becomes 103, 103, 103, and 104 cfu·mL^−1^, respectively, for the above samples, which is decreased by 10 times. The average recovery of *S. enteritidis* is between 81.83% and 110.56%, and the RSD values are all less than 10%, indicating that the newly created immunochromatography technique has a lot of potential to be employed with actual samples.

#### 5.2.3. MnO_2_ Nanostructure-Based Fluorescent Sensing

The MnO_2_ nanostructure exhibits high fluorescence quenching efficiency. Consequently, it is necessary to apply the MnO_2_ nanostructure and its composites in novel fluorescent sensors.

One potential indicator for evaluating the early post-slaughter freshness of aquatic products is the hypoxanthine concentration in them. Xu et al. synthesized a MnO_2_ in situ-coated upconversion nanoparticle (UCNP) composite (MnO_2_@UCNPs) *via* an improved sacrifice template method based on reducing KMnO_4_ by carbon. Utilizing the composite as the substrate, the highly efficient and sensitive hypoxanthine sensing platform was constructed based on hypoxanthine oxidation-induced MnO_2_ reduction and fluorescence turn-on [78] (Figure 7c). The synthesis of MnO_2_@UCNPs proceeded as follows: First, carbon-coated UCNPs (UCNPs@C) were synthesized *via* a one-step hydrothermal method. Subsequently, the MnO_2_@UCNP composite was yielded by in-situ reducing of a KMnO_4_ solution, utilizing UCNPs@C as the reducing agent and structure-directing agent concurrently. In the presence of a *p*-cresol oxidant and within a *p*-cresol concentration of 0.5–50 mg/L, the fluorescence intensity of the MnO_2_@UCNP composite was positively correlated with *p*-cresol concentration. The detection limit of the platform for *p*-cresol sensing was 0.14 mg/L. Specifically, the detection mechanism of hypoxanthine by MnO_2_@UCNP-based fluorescence platform is as follows: Under 980 nm laser excitation, UCNPs exhibit intense green and red peaks at approximately 550 nm and 665 nm, respectively. After encapsulating the MnO_2_ shell, the fluorescence intensity of the MnO_2_@UCNP composite becomes significantly weakened. The oxidation of hypoxanthine catalyzed by hypoxanthine oxidase yields reducing agents, including H_2_O_2_ and uric acid, which further reduce the MnO_2_ coating to Mn^2+^. The MnO_2_@UCNP composite progressively recovers its original fluorescence intensity with the gradual disappearance of the MnO_2_ shell. The designed fluorescent sensing platform demonstrates broad applications in the quality control of aquatic products containing hypoxanthine. Moreover, two kinds of fish (large yellow croaker and olive flounder), shrimp (*Trachypenaeus curvirostris*), and shellfish (scallops) samples were chosen as practical seafood samples. The hypoxanthine concentrations of the samples at 4°C in three post-mortem periods were obtained by the proposed nanosystem and the high performance liquid chromatography (HPLC) method. There exists no statistical difference between HPLC and the proposed method for determining the concentration of hypoxanthine in actual seafood samples (*p* > 0.05), suggesting that the proposed nanosystem could realize the accurate detection of hypoxanthine in various real seafoods with high efficiency and relatively low cost.

### 5.3. Biomedical Monitoring

Common biomolecules related to life safety include H_2_O_2_, glucose, Staphylococcus aureus, alkaline phosphatase, ascorbic acid, cysteine, uric acid, glutathione, acetaminophen and cancer biomarkers. Electrochemical sensing, electrochemiluminescence sensing, PEC sensing, colorimetric sensing, fluorescence sensing, photothermal sensing, microfluidic sensing and microcantilever sensing based on MnO_2_ nanostructures can all be employed for biomedical monitoring.

Applications of the MnO_2_ nanostructure and its composites in biomedical monitoring over the past five years have been summarized in Table 4, specifically covering the sensing mechanism, the structure and morphology of MnO_2_, the chemical composition of the substrate and analyte, the linear detection range and the minimum detection limit of analyte, and the corresponding reference.

#### 5.3.1. MnO_2_ Nanostructure-Based Electrochemical Sensing

MnO_2_ demonstrates exceptionally prominent advantages in novel electrochemical sensing, because it can reduce the overpotential of electrochemical reactions and enhance the current response of the system as an excellent redox couple.

**Figure 8:**
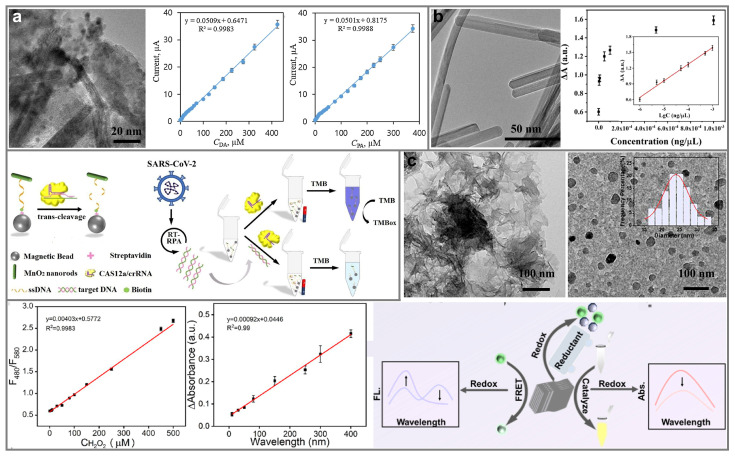
MnO_2_ nanostructure-based novel sensing for food-safety monitoring. (**a**) The detection of DA and PA based on electrochemical technology utilizing Pd@*α*-MnO_2_/G as the substrate: TEM image of Pd@*α*-MnO_2_/G and linear plots of *I_p_* vs. CDA and *I_p_* vs. CPA; (**b**) TEM image of MnO_2_ NRs, the linear calibration curve in the range of 1 ng/L to 1 μg/L, and SARS-CoV-2 detection utilizing *ss*DNA-MnO_2_-MB-based colorimetric technology; (**c**) detection of H_2_O_2_ based on dual-channel output signals including ratio-fluorescence and UV absorption utilizing MnO_2_/FPDA nanoparticle as the substrate: TEM images of MnO_2_ NSs and FPDA (inset: the size distribution), fluorescence and UV–vis of the FPDA/MnO_2_/OPD system at various H_2_O_2_ concentrations (FL: 0, 5, 10, 50, 80, 100, 150, 200, 250, 300, 450, and 500 μM; UV–vis: 0, 15, 30, 80, 100, 150, 200, 250, 300, 400, and 500 μM), and detection mechanism of the sensing platform.

MnO_2_/G was first synthesized *via* a one-step hydrothermal process, and Pd was subsequently doped onto the surface of MnO_2_/G through a two-step hydrothermal process, simultaneously filling cation vacancy and generating significant surface defects [92] (Figure 8a). The synthesized Pd@*α*-MnO_2_/G was applied to electrochemical-based detecting of biomolecule dopamine (DA) and paracetamol (PA). The electrochemically active surface area of Pd@*α*-MnO_2_/G was approximately two-times higher than that of *α*-MnO_2_/G, which proved that the Pd led to remarkable electrochemical performance. Meanwhile, the semicircle diameters of the electrodes followed the order of bare GCE (287.9 Ω) > reduced graphene oxide (211.2 Ω) > *α*-MnO_2_/G (136.2 Ω) > Pd@*α*-MnO_2_/G (86.7 Ω). Thus, Pd@*α*-MnO_2_/G exhibited the lowest value, confirming its faster electron transfer ability and better conductivity during the electrochemical reaction. It demonstrated a broad linear detection range for DA of 0.2–425 µM and a PA of 0.1–375 µM, an extremely low detection limit of 0.086 µM for DA and 0.059 µM for PA respectively, a high sensitivity of 0.0591 µA/µM and 0.0854 µA/µM for DA and PA, as well as excellent signal stability. Further analysis revealed that the abundant defects on Pd@*α*-MnO_2_/G significantly alter the electronic and crystalline structure of the prepared composite material, which further endow Pd@*α*-MnO_2_/G with a higher electroactive surface area, faster electron transfer capability and greater structural stability, thereby conferring outstanding electrochemical sensing performance. Moreover, the practicability of Pd@*α*-MnO_2_/G was verified by determining DA and PA in drinking water and human urine samples. The RSD values were 1.1% for DA and 1.8% for PA in drinking water, and 1.6% for DA and 2.5% for PA in urine. The recovery range of DA and PA in real samples is 97.24–102.8, which indicates the potential of the modified electrode for real-time applications.

#### 5.3.2. MnO_2_ Nanostructure-Based Colorimetric Sensing

The MnO_2_ nanostructure exhibits unique enzyme-like catalytic activity, and it is worth it to apply the MnO_2_ nanostructure and its composites in novel colorimetric sensors.

Huang et al. constructed a MnO_2_ nanozyme-mediated CRISPR-Cas12a-based colorimetric sensing system for SARS-CoV-2 detection [93] (Figure 8b). The MnO_2_ nanozyme was designed as follows: there exists a biotin group at one end and a carboxyl group at the other in single-stranded DNA (ssDNA); the biotin end strongly binds to streptavidin-coated magnetic beads (MBs), while the carboxyl end attaches to MnO_2_ nanorods, forming a MnO_2_ nanozyme (ssDNA-MnO_2_-MBs). The colorimetric sensing platform exhibited a good positive linear correlation between SARS-CoV-2 activity and solution color change within its concentration of 1 ng/L^−1^ μg/L and with a detection limit of 0.32 ng/L for SARS-CoV-2. The detection mechanism of the designed colorimetric sensing system for SARS-CoV-2 is as follows: CRISPR-Cas12a is activated upon adding SARS-CoV-2 target nucleic acid, initiating cleavage of the ssDNA linker and releasing MnO_2_ nanorods and MB units. Then, the TMB can be oxidized and catalyzed by MnO_2_ nanorods, causing a transformation of the solution color from pale yellow to blue. Moreover, SARS-E, hCov-HKU1-E and MERS-uPE-E pseudoviruses were constructed to investigate the specificity of the system. The three above-maintained pseudoviruses and SARS-CoV-2-E pseudovirus with a concentration of 100 copies/μL were analyzed using the MnO_2_ nanozyme-mediated CRISPRCas12a system. Only the SARS-CoV-2 pseudovirus caused a color change and was detected, while none of the other three pseudoviruses showed a noticeable signal change, indicating excellent selectivity. The designed colorimetric sensing system is characterized by simple operations and low cost, demonstrating its significant potential for molecular diagnostics in point-of-care testing.

#### 5.3.3. MnO_2_ Nanostructure-Based Fluorescent Sensing

It is worth it to apply the MnO_2_ nanostructure and its composites in novel fluorescent sensor-based biomedical monitoring.

The reducing substance plays a crucial role in various physiological processes and antioxidant defense in living organisms. Nevertheless, accurately detecting and distinguishing reducing substances remains a significant challenge due to potential interference in complex samples. Meiling Liu et al. developed a nanoplatform that consists of a MnO_2_ nanozyme/fluorescent polydopamine nanoparticle (MnO_2_/FPDA) for H_2_O_2_ detection, which features dual-channel output signals including ratio-fluorescence and UV absorption [107] (Figure 8c). Notably, the platform exhibited a detection limit of 3.18 μM and 10.67 μM in a H_2_O_2_ concentration range of 5–500 μM and 15–500 μM, utilizing the fluorescence and colorimetric channel respectively. Given the comparatively higher concentration of H_2_O_2_ in milk and cancer cells compared to other substances, the developed platform proves to be effective in quantifying H_2_O_2_ in such samples. To assess the practical utility of the platform, the standard addition method was employed, revealing a recovery rate of 94.6 to 102.7% with a relative standard deviation below 4.3%. These outcomes affirm the suitability of the method for H_2_O_2_ analysis in complex practical samples. The detecting mechanism of the MnO_2_/FPDA-based dual-platform sensor is as follows: The non-fluorescent reducing agent OPD can be oxidized to DAP with distinct yellow fluorescence catalyzed by MnO_2_ with oxidase-like activity. The fluorescence originating from FPDA can be quenched in the system containing FPDA as a fluorescent emitter and the MnO_2_ as a fluorescent acceptor. MnO_2_ can be converted to Mn^2+^ under the impact of a reducing agent, leading to the fluorescence recovery of FPDA, the fluorescence reduction of DAP, and a simultaneous shift in the UV absorption peak of DAP. The research provides crucial insights into developing a sensing platform capable of selectively detecting specific reducing species in real samples or deploying sensor arrays to distinguish multiple components within real samples. These advances pave the way for enhancing the analysis and screening of reductive substances in cellular and food samples.

MnO_2_ is a strong oxidant and can react non-specifically with various reducing agents in real samples, such as blood or food. Hence, there exists selectivity challenges of MnO_2_ sensing in complex matrices. In real samples, the interference challenges of MnO_2_ sensing mainly come from reducing small molecules in blood or urine, matrix coexisting substances in blood, and structural analogues of MnO_2_. Among them, reducing small molecules include ascorbic acid, dopamine, uric acid in blood and urine, and glutathione in living organisms, and matrix coexisting substances include proteins, fats, cells, etc., in the blood, as well as other small molecules and anions and cations in the sample. The following strategies can effectively improve the selectivity of MnO_2_ sensing in real samples: introduce highly specific biological enzymes or “probes” with high recognition ability into MnO_2_, specifically recognizing the target-like “keys and locks”, and element doping and defect engineering can alter the electronic structure and introduce oxygen vacancies in MnO_2_, thereby changing its adsorption and catalytic selectivity.

## 6. Conclusions and Outlook

In summary, with the application of the MnO_2_ nanostructure in environmental monitoring, food-safety monitoring and biomedical monitoring as the core, the sensing advantages and synthesis strategy of 0D, 1D, 2D and 3D MnO_2_ have been detailed, illustrated first with examples based on distinct dimensions. Secondly, the detecting advantages and detection mechanisms of MnO_2_ nanostructure-based novel sensing technologies have been systematically summarized. Moreover, applications of the MnO_2_ nanostructure across environmental monitoring, food-safety monitoring and biomedical monitoring using diverse sensing technologies have been summarized comprehensively. Undoubtedly, the development and widespread adoption of a novel sensor based on the MnO_2_ nanostructure have laid a solid foundation for a sensitive, reliable, rapid, simple and low-cost analysis of environmental pollutants, food additives and biomarkers.

With the rapid advancement of materials science, nanoscale synthesis and artificial intelligence, novel sensors based on the MnO_2_ nanostructure are poised to achieve broader and deeper research and applications in public health and ecological balance. Looking ahead, key research directions and development trends for novel sensors based on the MnO_2_ nanostructure can be summarized as follows:

### 6.1. Modification of MnO_2_ Nanostructure

Sensing performance can be further enhanced by surface modifications of MnO_2_, the surface coating of MnO_2_, compositing MnO_2_ with other materials, and the design of the morphology and microstructure of MnO_2_. Surface modifications of the MnO_2_ nanostructure with molecular imprinting or biorecognition elements, including enzymes, antibodies and aptamers, are beneficial for improving the specificity and selectivity of sensors. Coating the MnO_2_ nanostructure with inert shells, including silica and alumina, enhances the structural stability of the substrate and improves the performance consistency of the sensor. Fixing the MnO_2_ nanostructure onto flexible substrates improves mechanical flexibility and enhances the potential application for flexible sensing of MnO_2_. Compositing the MnO_2_ nanostructure with carbon materials, including carbon quantum dots and graphenes; conductive polymers, including PEDOT and PSS; as well as noble metals, including Au, Ag and Pt, significantly boosts substrate conductivity, further improving sensor efficiency and response times. Developing the MnO_2_ nanostructure with an ultrathin layered or a mesoporous structure increases the specific surface area and active site of the substrate, further improving the response time and selectivity of sensors.

### 6.2. Industrial Production of MnO_2_ Nanomaterial

In the field of MnO_2_ nanostructure-based novel sensing, the research focus should shift from the pursuit of novel morphology and extreme performance at the laboratory level to the establishment of industrial production processes with a controllable process, strong repeatability and appropriate cost. The synthesis methods of MnO_2_ include hydrothermal/solvothermal methods, chemical precipitation/redox methods, electrochemical precipitation methods, liquid phase stripping methods, template methods, biosynthesis methods, spray pyrolysis methods, etc., and the hydrothermal method is relatively simple. At present, the hydrothermal synthesis of MnO_2_ is usually carried out in the intermittent high-pressure reactor, while its uneven mass and heat transfer leads to large differences among batch products, and amplification production directly becomes difficult. In the future, a continuous flow microreactor can be utilized as a candidate for the industrial production of MnO_2_ *via* a hydrothermal strategy. This can not only realize the uniform nucleation and growth of MnO_2_ by accurately controlling the reaction parameters in the microchannel but can also combine with an online monitoring system to ensure the consistency of cross-batch product quality.

### 6.3. Development of Multimodal Sensors

MnO_2_ nanostructure-based multimodal sensors leverage diverse physicochemical properties of MnO_2_, including electrochemical activity, optical activity and redox properties, to simultaneously output multiple signal modes, including electrical signals, optical signals and thermal signals. The multimodal sensing platform offers significant advantages, including high reliability, a wide dynamic range and adaptability based on synergistic detection mechanisms. Specifically, a multi-signal output effectively reduces false alarms caused by a single-signal output, thereby enhancing sensor reliability. Since different signal types exhibit varying sensitivity to analyte concentrations, a multimodal sensor typically offers a broader linear range for analyte detection. Additionally, distinct types of signals may preferentially interact with different analytes, making a multimodal sensor suitable for analyzing complex real-world samples, such as blood, wastewater and food. Typical MnO_2_ nanostructure-based multimodal sensors include electrochemical–colorimetric dual-mode sensors, electrochemical–photothermal dual-mode sensors and electrochemical–mechanical dual-mode sensors. Taking the electrochemical–colorimetric bimodal sensor as an example, when detecting H_2_O_2_ or glucose, the electrochemical signal can be generated through a redox reaction between the substrate and analyte on the one hand, and the solution becomes transparent *via* the reduction of Mn^4+^ to Mn^2+^ on the other hand. Consequently, the sensor can provide not only quantitative concentration data *via* electrochemical sensing but also semi-quantitative concentration data through colorimetric sensing.

### 6.4. Development of Intelligent Sensors

The intellectualization of a MnO_2_ nanostructure-based sensor involves endowing the sensor with automated functions, including adaptability, self-diagnosis and data interaction through various strategies, including intelligent signal processing and algorithms, as well as IoT integration. Specifically, detection signal processing in real time, noise filtering and conducting baseline calibration can be realized by integrating a microprocessor within the MnO_2_ nanostructure-based sensor. Moreover, the signal-to-noise ratio can be reduced and the detection accuracy can be improved by machine learning-assisted detection signal analysis. The uploading of sensing data to the cloud, and real-time transmission and remote monitoring of detection data can be conducted through a Bluetooth module. Intelligent sensors find widespread applications in healthcare, environmental monitoring and industrial safety surveillance. For instance, real-time wound infection monitoring and diabetes management can be conducted through analyzing H_2_O_2_ and glucose by MnO_2_ nanostructure-based intelligent sensors. Specifically, the detecting signal intensity and glucose concentration in MnO_2_-based sensing exhibits a non-linear relationship, especially at low and high concentrations. It is inaccurate to utilize a traditional linear regression strategy to fit the correlation between detecting signal intensity and glucose concentration. Conversely, machine learning can perform non-linear regression modeling on calibration data, which can significantly improve the prediction accuracy of glucose concentrations. Abreu et al. reported a MnO_2_-based enzyme-modified screen-printed electrode for the electrochemical detection of glucose in serum. The sensor has a linear range of 0.75–40 mM and a detection limit of 0.078 mM. On the basis of this, the researchers utilized a variety of machine learning algorithms to build a model for “glucose concentration–signal intensity”. The decision tree algorithm accurately predicts the calibration parameter and acquires a coefficient determination exceeding 0.9, and the multi-layer perceptron model effectively predicted the glucose concentration with a determination coefficient of 0.828, which fully demonstrated the reliability of machine learning-assisted electrochemical sensing for glucose detection. Machine learning technology can also be integrated with embedded systems to achieve portable, real-time diabetes detection. Researchers combined micro-machine learning with an electronic nose of metal oxide sensors for real-time detection of diabetes. The XGBoost algorithm has a detection accuracy of 95%, and the deep neural network and 1D convolutional neural network have a detection efficiency of 94.44%, which provides important technical guidance for the development of non-embedded diabetes diagnosis equipment. Future advancements are anticipated through integrating bioengineering, artificial intelligence and microelectronics to drive the development of next-generation MnO_2_ nanostructure-based intelligent sensing systems.

## Figures and Tables

**Table 1:** MnO_2_ nanostructure-based novel sensing for air pollutant monitoring.

Nanostructure	MnO_2_ Morphology	Substrate	Analyte	LOD	Response	Response Time	Reference
0D	nanoparticle	PANI@MnO_2_@rGO	NH_3_	0.21 ppm	15.56% under 50 ppm	6–10 s	[64]
	nanoparticle	graphene oxide@ MnO_2_	NO_2_	385 ppm	16.3% under 1 ppm		[28]
1D	nanowire	Ni-MnO_2_/ Ti_3_C_2_T_x_·MXene	NH_3_	0.23 ppm		45 s	[65]
	nanorod	Co-MnO_2_@ MnCo_2_O_4.5_	triethylamine		340% under 50 ppm	9 s	[66]
2D	nanosheet	MXene·Ti_3_C_2_T_X_@ NiCo_2_O_4_@MnO_2_	CH_3_COOH	0.05 ppm	12.5% under 20 ppm	130 s	[67]
	nanosheet	NiO/MnO_2_@NiO	allyl mercaptan	15 ppb	56.69% under 40 ppm	115 s	[1]
3D	nanoflower	MnO_2_@Ti_3_C_2_T_x_	glyoxaldhyde	20 ppm	52% under 20 ppm	134 s	[57]
	nanonest	MnO_2_/TiO_2_	triethylamine	12 ppb	6.8% under 100 ppm		[68]

**Table 2:** MnO_2_ nanostructure-based novel sensing for pollutant monitoring in water quality and soil.

Nanostructure	Sensing Mechanism	MnO_2_ Morphology	Substrate	Analyte	Linear Detection Range	Minimum Detection Limit	Reference
0D	electrochemical sensing	nanoparticle	vancomycin@ MnO_2_ nanozyme	staphylococcus aureus		10 cfu/mL	[27]
	electrochemical sensing	nanoparticle	PANI@ MnO_2_	nitrite	CV: 0.1–10 mM CA: 19.98–732.17 μM	CV: 4.38 μM CA: 1.08 μM	[29]
	colorimetric sensing	nanoparticle	CuO@MnO_2_ nanozyme	2-mercaptobenzothiazole	0.1–15 μM	5.9 nM	[26]
	colorimetric sensing	nanoparticle	crystalline MnO_2_	Se^4+^	10–600 μg/L	1.8 μg/L	[69]
	colorimetric sensing	nanoparticle	conjugated polymer nanoparticles@ MnO_2_	organo-phosphorus pesticides		0.025 ng/mL	[70]
1D	electrochemical sensing	nanorod	Cu-MOF/ rGO/CuO@ *α*-MnO_2_	resorcinol	0.2–22 μM	0.2 μM	[71]
	colorimetric sensing	nanorod	PANI@ MnO_2_ nanozyme	organo-phosphorus pesticides	0.50–50 μM	0.39 μM	[72]
2D	PEC sensing	nanosheet	Ag_2_S quantum dot@MnO_2_	Cr^6+^	100 pM–30 μM	6.46 pM	[40]
	colorimetric sensing	nanosheet	carbon dot@MnO_2_	Hg^2+^	0.01–0.1 μM	0.01 μM	[33]
	colorimetric sensing	nanosheet	ultrathin MnO_2_ nanomembrane	tetracycline antibiotics	2–225 μM	0.4 μM	[73]
	fluorescence sensing	nanosheet	branched poly(ethyleneimine) carbon quantum dots@MnO_2_ nanosheet	malachite green	0.2–14 μM	0.06 μM	[74]
	fluorescence sensing	nanosheet	TPB-DMTP@ S-CDs/MnO_2_	organo-phosphorus pesticides	1 × 10^−6^–12 μg/mL	7.9 × 10^−7^ μg/mL	[75]
3D	fluorescence sensing	octahedron	UiO-66-NH_2_/ MnO_2_	organo-phosphorus pesticides	1.0 × 10^−7^–10 mg/L	8.9 × 10^−8^ mg/L	[76]
	QCM sensing	sea urchin	GCN@ MnO_2_	chloramphenicol		11 μM	[18]

**Table 3:** MnO_2_ nanostructure-based novel sensing for food-safety monitoring.

Nanostructure	Sensing Mechanism	MnO_2_ Morphology	Substrate	Analyte	Linear Detection Range	Minimum Detection Limit	Reference
0D	electrochemical sensing	nanoparticle	ZnO@ MnO_2_-rGO	antioxidant molecules	hydroquinone: 0.008–10 µM and 10–350 µM monotert-butyl hydroquinone: 0.008–10 µM and 10–320 µM	hydroquinone: 0.0011 µM monotert-butyl hydroquinone: 0.0012 µM	[77]
	fluorescence sensing	nanoparticle	upconversion nanoparticles@ MnO_2_	pseu- dopurine	0.5–50 mg/L	0.14 mg/L	[78]
	fluorescence sensing	nanoparticle	carbon quantum dots@MnO_2_ nanoparticles	nitrite			[79]
	colorimetric sensing	nanoparticle	PDA/MnO_2_ nanoenzyme	dithio- carbamate		0.63 ng/mL	[80]
1D	electrochemical sensing	nanotube	MnO_2_ nanotubes@ layered double hydroxide nanocages	H_2_O_2_	1 × 10^–3^–4 mmol/L 4–8 mmol/L	0.26 µmol/L	[81]
2D	electrochemical sensing	nanosheet	graphene@ MnO_2_	organo-phosphorus pesticide	3–4000 ng/mL	1.2 ng/mL	[82]
	electrochemical sensing	nanosheet	MnO_2_ nanoenzyme	organo-phosphorus pesticide		0.025 ng/mL	[83]
	colorimetric sensing	nanosheet	MnO_2_ nanoenzyme	shigella flexneri	10^3^–10^7^ cfu/mL	10^3^ cfu/mL	[84]
	colorimetric sensing	nanosheet	zein/MnO_2_ composite	H_2_O_2_ and lactic acid		H_2_O_2_: 7.2 × 10^−4^ mol/L lactic acid: 7.5 × 10^−4^ mol/L	[85]
	fluorescence sensing	nanosheet	okra carbon nanoparticles@ MnO_2_ nanosheets	DDVP	4–120 μg/L	1.2 μg/L	[86]
	fluorescence sensing	nanosheet	Au nanoclusters@ MnO_2_ nanosheets	methyl parathion	0.005–200 ng/mL	3.1 pg/mL	[87]
	fluorescence sensing	nanosheet	Ru@UiO-66@ MnO_2_ nanosheets/ thiamine	DDVP and chlorpyrifos		DDVP: 9.99 × 10^−6^ μg/mL chlorpyrifos: 9.99 × 10^−5^ μg/mL	[88]
3D	colorimetric sensing	nanoflower	MnO_2_ nanoenzyme	aflatoxin M1	6–160 ng/L	2.1 ng/L	[89]
	fluorescence sensing	nanoflower	MnO_2_ nanoflower	ochratoxin A	0.05–33.35 ng/mL	0.069 ng/mL	[90]

**Table 4:** MnO_2_ nanostructure-based novel sensing for biomedical monitoring.

Nanostructure	Sensing Mechanism	MnO_2_ Morphology	Substrate	Analyte	Linear Detection Range	Minimum Detection Limit	Reference
0D	fluorescence sensing	nanoparticle	Fe_3_O_4_@ Polydopamine @MnO_2_	disease marker alkaline phosphatase	0.05–40 U/L	0.045 U/L	[91]
	photothermal sensing	nanoparticle	Au@MnO_2_ core-shell structure	disease marker alkaline phosphatase	2.0–50 U/L	0.75 U/L	[17]
1D	electrochemical sensing	nanowire	phage templated MnO_2_ nanowires	glucose in blood sugar	5 μM–2 mM	1.8 μM	[38]
	electrochemical sensing	nanorod	Pd-*α*-MnO_2_@ graphene	neuro- transmitter dopamine, hepatotoxic and nephrotoxic agent, aceta- minophen	dopamine: 0.2–425 µM acetaminophen: 0.1–375 µM	dopamine: 0.0591 µM acetaminophen: 0.0854 µM	[92]
	electrochemical sensing	nanorod	rGO@MOFs@ *α*-MnO_2_	acetylcholine	0.1 µM–3 mM	5 nM	[35]
	fluorescence sensing	nanorod	MnO_2_ nanoenzyme	SARS-CoV-2			[93]
2D	electrochemical sensing	nanosheet	MnO_2_ nanoenzyme	Alzheimer’s disease biomarker *β*-amyloid-like protein	0.01–500 nM	0.44 pmol/L	[94]
	EL sensing	nanosheet	MnO_2_@ polydopamine	immune biological marker *α*-fetoprotein	0.01 pg/mL–5 ng/mL	10.7 fg/mL	[95]
	PEC sensing	nanosheet	MnO_2_@ Co_3_O_4_/Ti	superoxide anion in mitochondria	0.1–50,000 nM	0.025 nM	[41]
	colorimetric sensing	nanosheet	carbon quantum dots@MnO_2_ nanosheets	oxalate in urine	1–50 μM	690 nM	[96]
	colorimetric sensing	nanosheet	upconversion nanoparticles @MnO_2_	glutathione in cells		0.9 μM	[97]
	fluorescence sensing	nanosheet	dopamine accumulation @MnO_2_	serum disease marker alkaline phosphatase	1–80 U/L	0.34 U/L	[98]
	fluorescence sensing	nanosheet	Au nanoclusters @MnO_2_ nanosheets	cardiac marker troponin T	0–16 ng/mL	0.037 ng/mL	[99]
	photothermal sensing	nanosheet	Au nanorods @MnO_2_ nanosheets	melanoma biomarker tyrosinase	1.0–70 U/mL	0.34 U/mL	[100]
	QCM sensing	nanosheet	polypyrrole/ MnO_2_@ cellulose	G-series nerve agent methyl phosphonic acid dimethyl ester			[101]
3D	electrochemical sensing	nanoflower	Co-MnO_2_@ rGO-CNT	cancer biomarker H_2_O_2_	0.2 μM–18.0 mM	66.7 nM	[102]
	PEC sensing	nanomotor	MnO_2_-carbon nanobottle	microRNA	100 fM–100 nM	100 fM	[103]
	colorimetric sensing	nanoball	Ru-MnO_2_ nanoenzyme	cancer biomarker H_2_S and cystathionine-*β*-lyase	H_2_S: 0.1–70 μM cystathionine γ-lyase: 1–110 µM	H_2_S: 0.098 μM cystathionine γ-lyase: 0.95 µM	[104]
	colorimetric sensing	hollow cube	H-MnO_2_ nanoenzyme	liver function biomarkers		aspartate aminotransferase: 4.9 U/L alanine aminotransferase: 3.6 U/L alkaline phosphatase: 0.99 U/L	[56]
	colorimetric sensing	nanoflower	carbon quantum dots@MnO_2_ nanoblossoms	glutathione in cells	2.0–200 μM	0.558 μM	[105]
	fluorescence sensing	nanoflower	CaF_2_@MnO_2_	serum uric acid	0.1–30 μM	0.039 μM	[106]

## Data Availability

No new data were created or analyzed in this study. Data sharing is not applicable to this article.

## References

[1] Li C., Choi P.G., Masuda Y. (2022). Highly sensitive and selective gas sensors based on NiO/MnO_2_@NiO nanosheets to detect allyl mercaptan gas released by humans under psychological stress. Adv. Sci..

[2] Vågerö D., Cederström A., van den Berg G.J. (2022). Food abundance in men before puberty predicts a range of cancers in grandsons. Nat. Commun..

[3] Anderson W., Taylor C., McDermid S., Ilboudo-Nébié E., Seager R., Schlenker W., Cottier F., De Sherbinin A., Mendeloff D., Markey K. (2021). Violent conflict exacerbated drought-related food insecurity between 2009 and 2019 in sub-Saharan Africa. Nat. Food.

[4] Cattaneo I., Astuto M.C., Binaglia M., Devos Y., Dorne J.L.C., Agudo A.F., Dumont A.F., Garcia-Vello P., Kass G.E., Lanzoni A. (2023). Implementing New Approach Methodologies (NAMs) in food safety assessments: Strategic objectives and actions taken by the European Food Safety Authority. Trends Food Sci. Technol..

[5] Camargo J.R., Baccarin M., Raymundo-Pereira P.A., Campos A.M., Oliveira G.G., Fatibello-Filho O., Oliveira O.N., Janegitz B.C. (2018). Electrochemical biosensor made with tyrosinase immobilized in a matrix of nanodiamonds and potato starch for detecting phenolic compounds. Anal. Chim. Acta.

[6] Kumar S., Bukkitgar S.D., Singh S., Pratibha, Singh V., Reddy K.R., Shetti N.P., Venkata Reddy C., Sadhu V., Naveen S. (2019). Electrochemical sensors and biosensors based on graphene functionalized with metal oxide nanostructures for healthcare applications. ChemistrySelect.

[7] Mahlangu W.B., Maseko B.R., Mongadi I.L., Makhubela N., Ncube S. (2023). Quantitative analysis and health risk assessment of bisphenols in selected canned foods using the modified QuEChERS method coupled with gas chromatography-mass spectrometry. Food Packag. Shelf Life.

[8] Abdullahi A.B., Ismail S., Alshana U. (2023). Edible oil-based switchable-hydrophilicity solvent liquid–liquid microextraction for the determination of lead in food samples using flame-atomic absorption spectrometry. J. Food Compos. Anal..

[9] Huang Y.-H., Hirose D., Minami J., Wang M.-J., Takamura Y. (2022). Fabrication and characterizations of axial view liquid electrode plasma atomic emission spectrometry for the sensitive determination of trace zinc, cadmium, and lead. Anal. Chem..

[10] Karbelkar A.A., Reynolds E.E., Ahlmark R., Furst A.L. (2021). A microbial electrochemical technology to detect and degrade organophosphate pesticides. ACS Cent. Sci..

[11] Chamorro-Garcia A., Parolo C., Ortega G., Idili A., Green J., Ricci F., Plaxco K.W. (2022). The sequestration mechanism as a generalizable approach to improve the sensitivity of biosensors and bioassays. Chem. Sci..

[12] Ripolles-Avila C., Martínez-Garcia M., Capellas M., Yuste J., Fung D.Y., Rodríguez-Jerez J.J. (2020). From hazard analysis to risk control using rapid methods in microbiology: A practical approach for the food industry. Compr. Rev. Food Sci. Food Saf..

[13] Wang Y., Zhao G., Li X., Liu L., Cao W., Wei Q. (2018). Electrochemiluminescent competitive immunosensor based on polyethyleneimine capped SiO_2_ nanomaterials as labels to release Ru (bpy) 32+ fixed in 3D Cu/Ni oxalate for the detection of aflatoxin B1. Biosens. Bioelectron..

[14] Ge L., Liu Q., Hao N., Kun W. (2019). Recent developments of photoelectrochemical biosensors for food analysis. J. Mater. Chem. B.

[15] Priyadarshi R., Ezati P., Rhim J.-W. (2021). Recent advances in intelligent food packaging applications using natural food colorants. ACS Food Sci. Technol..

[16] Sharma A.S., Ali S., Sabarinathan D., Murugavelu M., Li H., Chen Q. (2021). Recent progress on graphene quantum dots-based fluorescence sensors for food safety and quality assessment applications. Compr. Rev. Food Sci. Food Saf..

[17] Li J., Liu X.-P., Ye W.-Q., Xu Z.-R. (2023). Photothermal visual sensing of alkaline phosphatase based on the etching of Au@ MnO_2_ core-shell nanoparticles. J. Colloid Interface Sci..

[18] Shaheen A., Idrees F., Butt F.K., Mujahid A., Afzal A., Ullah S., Asim T., Khan W.S., Bajwa S.Z. (2025). Synthesis and integration of sea urchin-like MnO_2_-GCN nanocomposite with imprinted polymers for mass-sensitive detection of chloramphenicol in water. Compos. Commun..

[19] Shetti N.P., Mishra A., Basu S., Mascarenhas R.J., Kakarla R.R., Aminabhavi T.M. (2020). Skin-patchable electrodes for biosensor applications: A review. ACS Biomater. Sci. Eng..

[20] Dakshayini B., Reddy K.R., Mishra A., Shetti N.P., Malode S.J., Basu S., Naveen S., Raghu A.V. (2019). Role of conducting polymer and metal oxide-based hybrids for applications in ampereometric sensors and biosensors. Microchem. J..

[21] Tehseen B., Rehman A., Rahmat M., Bhatti H.N., Wu A., Butt F.K., Naz G., Khan W.S., Bajwa S.Z. (2018). Solution growth of 3D MnO_2_ mesh comprising 1D nanofibres as a novel sensor for selective and sensitive detection of biomolecules. Biosens. Bioelectron..

[22] Samdani J., Samdani K., Kim N.H., Lee J.H. (2017). A new protocol for the distribution of MnO_2_ nanoparticles on rGO sheets and the resulting electrochemical performance. Appl. Surf. Sci..

[23] Hiremath S.D., Banerjee M., Chatterjee A. (2022). Review of 2D MnO_2_ nanosheets as FRET-based nanodot fluorescence quenchers in chemosensing applications. ACS Appl. Nano Mater..

[24] Sohal N., Maity B., Shetti N.P., Basu S. (2021). Biosensors based on MnO_2_ nanostructures: A review. ACS Appl. Nano Mater..

[25] Chen J., Wang C., Qin X., Yang X., Yang C., Nie H., Chen H., Li H. (2023). Manganese dioxide nanostructures: Design, synthesis and conceptualization for developing innovative sensors in reporting environmental risk factors. Coord. Chem. Rev..

[26] Zhang Y., Wang D., Liu G., Xiang Y., Wang K., Shuang S., Dong C. (2024). All-in-one high performance CuO@ MnO_2_ nanozymes: Dual-signal channel 2-mercaptobenzothiazole sensing and device-free oxidative degradation. Sens. Actuators B Chem..

[27] Ma Y., Lin X., Xue B., Luan D., Jia C., Feng S., Bian X., Zhao J. (2024). Ultrasensitive and highly selective detection of Staphylococcus aureus at the single-cell level using bacteria-imprinted polymer and vancomycin-conjugated MnO_2_ nanozyme. Anal. Chem..

[28] Alouani M.A., Casanova-Chafer J., de Bernardi-Martin S., Garcia-Gomez A., Vilanova X., Llobet E. (2025). A NO_2_ Sensitive MnO_2_/Graphene Oxide Composite Based Gas Sensor. Chemosensors.

[29] Qiu Y., Qu K. (2022). Binary organic-inorganic nanocomposite of polyaniline-MnO_2_ for non-enzymatic electrochemical detection of environmental pollutant nitrite. Environ. Res..

[30] Zou W., Liu Y., Li R., Guo R. (2022). Ingenious multifunctional MnO_2_ quantum dot nanozymes with superior catechol oxidase-like activity for highly selective sensing of redox-active dopamine based on an interfacial passivation strategy. ACS Sustain. Chem. Eng..

[31] Qu A., Chen Q., Sun M., Xu L., Hao C., Xu C., Kuang H. (2024). Sensitive and selective dual-mode responses to reactive oxygen species by chiral manganese dioxide nanoparticles for antiaging skin. Adv. Mater..

[32] Phong N.H., Vu H.X.A., Van Hop N., Quyen N.D.V., Hai H.V.M., Luyen N.D., Lieu P.K., Khieu D.Q. (2023). Simultaneous determination of chloramphenicol and tinidazole by electrochemical analysis using MnO_2_/electrochemically reduced graphene oxide modified electrode. J. Sci. Adv. Mater. Devices.

[33] Thakuri A., Bhosle A.A., Hiremath S.D., Banerjee M., Chatterjee A. (2024). A carbon dots-MnO_2_ nanosheet-based turn-on pseudochemodosimeter as low-cost probe for selective detection of hazardous mercury ion contaminations in water. J. Hazard. Mater..

[34] Chen Y., Gong C., Chen K., Wang Z., He M., Wang P., Chen K., Jiao Y., Yang Y. (2024). G-quadruplex DNA-based colorimetric biosensor for the ultrasensitive visual detection of strontium ions using MnO_2_ nanorods as oxidase mimetics. Microchim. Acta.

[35] Ashraf G., Asif M., Aziz A., Iftikhar T., Zhong Z.-T., Zhang S., Liu B., Chen W., Zhao Y.-D. (2022). Advancing interfacial properties of carbon cloth via anodic-induced self-assembly of MOFs film integrated with α-MnO_2_: A sustainable electrocatalyst sensing acetylcholine. J. Hazard. Mater..

[36] Yang M., Wu Z., Wang X., Yin Z., Tan X., Zhao J. (2022). Facile preparation of MnO_2_–TiO_2_ nanotube arrays composite electrode for electrochemical detection of hydrogen peroxide. Talanta.

[37] Xukeer A., Li J. (2025). Construction of ZnO/MnO_2_ heterostructure for enhanced ammonia sensitive performances at room temperature. Appl. Surf. Sci..

[38] Han L., Shao C., Liang B., Liu A. (2016). Genetically engineered phage-templated MnO_2_ nanowires: Synthesis and their application in electrochemical glucose biosensor operated at neutral pH condition. ACS Appl. Mater. Interfaces.

[39] Miguel V.M., Rodrigues M.P.d.S., Braga A.H., de Torresi S.I.C. (2022). MnO_2_ nanowires decorated with Au nanoparticles for plasmon-enhanced electrocatalytic detection of H_2_O_2_. ACS Appl. Nano Mater..

[40] Zhao L., Chen H., Tang Y., Li P., Zhu X., Liu J., Liu M., Zhang Y., Yao S. (2023). Ag_2_S QDs integration with MnO_2_ nanosheets for the sensitive detection of Cr (VI) via the redox reaction induced photoelectrochemical variation. Anal. Chim. Acta.

[41] Zhao D., Geng C., Liu X., Jin X., Zhao Z., Liu Y., Alwarappan S. (2023). Photoelectrochemical detection of superoxide anions released from mitochondria in HepG2 cells based on the synergistic effect of MnO_2_@ Co_3_O_4_ core-shell pn heterojunction. Biosens. Bioelectron..

[42] Su L., Zhong J., Xu J., Wu H., Zhang Z., Xiong Y. (2022). Sunlight-assisted environmentally-friendly synthesis of graphene-like δ-MnO_2_ nanosheets for colorimetric sensing. FlatChem.

[43] Xie L., He L., Fu Y., Wei Y., Zhang K., Chen M. (2025). A three-site recognition cytosensor for accurate detection of circulating tumor cells based on novel branched Pt Au nanospheres and MnO_2_-GO-Au nanosheets. Microchim. Acta.

[44] Zhao D., Li B., Zhao Z., Ren Z., Zhu X., Liu X., Liu X. (2025). Detection of Hela cell mitochondria released superoxide anions with photoelectrochemically driven sensor based on core-shell Al-doped MnO_2_@ NiCo-metal organic framework nanosheet array. J. Power Sources.

[45] Yang D.-P., Guo W., Cai Z., Chen Y., He X., Huang C., Zhuang J., Jia N. (2018). Highly sensitive electrochemiluminescence biosensor for cholesterol detection based on AgNPs-BSA-MnO_2_ nanosheets with superior biocompatibility and synergistic catalytic activity. Sens. Actuators B Chem..

[46] Choi C.A., Lee J.E., Mazrad Z.A.I., In I., Jeong J.H., Park S.Y. (2018). Redox-and pH-responsive fluorescent carbon nanoparticles-MnO_2_-based FRET system for tumor-targeted drug delivery in vivo and in vitro. J. Ind. Eng. Chem..

[47] Liu J., Meng L., Fei Z., Dyson P.J., Jing X., Liu X. (2017). MnO_2_ nanosheets as an artificial enzyme to mimic oxidase for rapid and sensitive detection of glutathione. Biosens. Bioelectron..

[48] Li Q., Xia Y., Wan X., Yang S., Cai Z., Ye Y., Li G. (2020). Morphology-dependent MnO_2_/nitrogen-doped graphene nanocomposites for simultaneous detection of trace dopamine and uric acid. Mater. Sci. Eng. C.

[49] Çakıroğlu B., Demirci Y.C., Gökgöz E., Özacar M. (2019). A photoelectrochemical glucose and lactose biosensor consisting of gold nanoparticles, MnO_2_ and g-C_3_N_4_ decorated TiO_2_. Sens. Actuators B Chem..

[50] Jiao L., Zhang L., Du W., Li H., Yang D., Zhu C. (2018). Hierarchical manganese dioxide nanoflowers enable accurate ratiometric fluorescence enzyme-linked immunosorbent assay. Nanoscale.

[51] Wan X., Yang S., Cai Z., He Q., Ye Y., Xia Y., Li G., Liu J. (2019). Facile synthesis of MnO_2_ nanoflowers/N-doped reduced graphene oxide composite and its application for simultaneous determination of dopamine and uric acid. Nanomaterials.

[52] Ge J., Cai R., Chen X., Wu Q., Zhang L., Jiang Y., Cui C., Wan S., Tan W. (2019). Facile approach to prepare HSA-templated MnO_2_ nanosheets as oxidase mimic for colorimetric detection of glutathione. Talanta.

[53] Öner M., Demir C., Çetin G., Bakırdere S. (2023). Development of a rapid and efficient analytical method for trace lead determination: Manganese dioxide nanoflower based dispersive solid-phase extraction. Measurement.

[54] Li G., Qi X., Xiao Y., Zhao Y., Li K., Xia Y., Wan X., Wu J., Yang C. (2022). An efficient voltammetric sensor based on graphene oxide-decorated binary transition metal oxides Bi_2_O_3_/MnO_2_ for trace determination of lead ions. Nanomaterials.

[55] Wang L., Huo X., Jiang F., Xi X., Li Y., Lin J. (2023). Dual-functional manganese dioxide nanoclusters for power-free microfluidic biosensing of foodborne bacteria. Sens. Actuators B Chem..

[56] Li S., Chen Z., Yang F., Yue W. (2023). Self-template sacrifice and in situ oxidation of a constructed hollow MnO_2_ nanozymes for smartphone-assisted colorimetric detection of liver function biomarkers. Anal. Chim. Acta.

[57] Yao Y., Li Z., Han Y., Xie L., Zhao X., Zhu Z. (2023). Fabrication and characterization of a MnO_2_/Ti_3_C_2_Tx based gas sensor for highly sensitive and selective detection of lung cancer marker hexanal. Chem. Eng. J..

[58] Shetti N.P., Nayak D.S., Reddy K.R., Aminabhvi T.M. (2019). Graphene–clay-based hybrid nanostructures for electrochemical sensors and biosensors. Graphene-Based Electrochemical Sensors for Biomolecules.

[59] Baranwal J., Barse B., Gatto G., Broncova G., Kumar A. (2022). Electrochemical sensors and their applications: A review. Chemosensors.

[60] Ning Z., Chen M., Wu G., Zhang Y., Shen Y. (2021). Recent advances of functional nucleic acids-based electrochemiluminescent sensing. Biosens. Bioelectron..

[61] Suo Z., Yu T., Xu Y., Ren W., Liu Y., Wei M., Jin H., He B., Zhao R. (2025). Research progress of photoelectrochemical sensors in food detection. Food Res. Int..

[62] Liu B., Zhuang J., Wei G. (2020). Recent advances in the design of colorimetric sensors for environmental monitoring. Environ. Sci. Nano.

[63] Brzechwa-Chodzyńska A., Drożdż W., Harrowfield J., Stefankiewicz A.R. (2021). Fluorescent sensors: A bright future for cages. Coord. Chem. Rev..

[64] Umar A., Akbar S., Kumar R., Ahmed F., Ansari S.A., Ibrahim A.A., Alhamami M.A., Almehbad N., Algadi H., Almas T. (2024). Unveiling the potential of PANI@ MnO_2_@ rGO ternary nanocomposite in energy storage and gas sensing. Chemosphere.

[65] Elancheziyan M., Singh M., Bhuvanendran N., Won K. (2025). Ni-doped MnO_2_/Ti_3_C_2_Tx MXene nanocomposite for highly sensitive electrochemical ammonia gas sensing at room temperature. J. Alloys Compd..

[66] Hu X., Chen H., Zhang K., Tian D., Cao Y., Zhu Z. (2024). Synergistic enhancement of triethylamine sensing performances via oxygen vacancy-rich Co-doped MnO_2_@MnCo_2_O_4.5_ nanorods/nanosheets. J. Mater. Chem. C.

[67] Akhtar A., Sadaf S., Zhou R., Ling Q., Luo S., Han M., Di W., Liu J., Chen D., Chu X. (2025). Highly responsive and stable room temperature acetic acid gas sensor based on nano-composite of MXene Ti_3_C_2_TX-NiCo_2_O_4_-MnO_2_. J. Alloys Compd..

[68] Jin Q., Wen W., Wang Z.-X., Wang R.-H., Zheng S., Ye Z., Wu J.-M. (2022). Nanoarchitectonics of nest-like MnO2/TiO2 thin film for triethylamine sensing. Sens. Actuators B Chem..

[69] Wang B., Pu S., Ma B., Zou X., Xiong Q., Hou X., Xu K. (2024). Selenium hydride-induced oxidase-like activity inhibition of amorphous/crystalline manganese dioxide: Colorimetric assay for selenium detection. Anal. Chem..

[70] Li Q., Zhang J., Wang Y., He Z., Xu Z., Zhang M., Cheng Y. (2025). A ratiometric fluorescence and visual sensor based on conjugated polymer nanoparticles@ MnO_2_ probe for organophosphorus pesticides detection. Talanta.

[71] Iftikhar T., Xu Y., Aziz A., Ashraf G., Li G., Asif M., Xiao F., Liu H. (2021). Tuning electrocatalytic aptitude by incorporating α-MnO_2_ nanorods in Cu-MOF/rGO/CuO hybrids: Electrochemical sensing of resorcinol for practical applications. ACS Appl. Mater. Interfaces.

[72] Yang C.-L., Yu L.-H., Pang Y.-H., Shen X.-F. (2024). A colorimetric sensing platform with smartphone for organophosphorus pesticides detection based on PANI-MnO_2_ nanozyme. Anal. Chim. Acta.

[73] Wu S.-T., Su H.-Q., Xiao Q.-X., Qiu Z.-Y., Huang G.-Q., He M.-N., Ge Y., Wang C.-H., Lin Y.-W. (2024). Design of bifunctional ultrathin MnO_2_ nanofilm with laccase-like activity for sensing environmental pollutants containing phenol groups. J. Hazard. Mater..

[74] Mu X., Liu X., Ye X., Zhang W., Li L., Ma P., Song D. (2022). Branched poly (ethylenimine) carbon dots-MnO_2_ nanosheets based fluorescent sensory system for sensing of malachite green in fish samples. Food Chem..

[75] Yuan L., Tian X., Fan Y., Sun Z., Zheng K., Zou X., Zhang W. (2023). TPB-DMTP@ S-CDs/MnO_2_ fluorescence composite on a dual-emission-capture sensor module for fingerprint recognition of organophosphorus pesticides. Anal. Chem..

[76] Yuan L., Gan Z., Fan Y., Ding F., Xu X., Chen X., Zou X., Zhang W. (2022). Thermal-controlled active sensor module using enzyme-regulated UiO-66-NH_2_/MnO_2_ fluorescence probe for total organophosphorus pesticide determination. J. Hazard. Mater..

[77] Movahed V., Arshadi L., Ghanavati M., Nejad E.M., Mohagheghzadeh Z., Rezaei M. (2023). Simultaneous electrochemical detection of antioxidants Hydroquinone, Mono-Tert-butyl hydroquinone and catechol in food and polymer samples using ZnO@ MnO_2_-rGO nanocomposite as sensing layer. Food Chem..

[78] Cao Y., Song Y., Wei T., Feng T., Li M., Xue C., Xu J. (2024). MnO_2_ in-situ coated upconversion nanosystem for turn-on fluorescence detection of hypoxanthine in aquatic products. Food Chem..

[79] Peng B., Wang C., He X., Ma Y., Zhou M., Ma X., Zhao S., Fang Y. (2023). A smartphone-assisted ratiometric colorimetric and fluorescent probe for triple-mode determination of nitrite based on MnO_2_ nanoparticles and carbon quantum dots. Food Chem..

[80] Guo J., Zhao F., Yue Z., Lei Z. (2025). Acetylcholinesterase-free colorimetric sensing platform for carbosulfan detection based on hollow PDA/MnO_2_ nanozyme. Food Chem..

[81] Zhang Y., Jin Y., Yuan X., Zhao S., Ye J., Xue K., Hu J., Xiong X. (2023). Layered bimetallic hydroxide nanocage assembled on MnO_2_ nanotubes: A hierarchical porous sugar gourd-like electrocatalyst for the sensitive detection of hydrogen peroxide in food. Food Chem..

[82] Liu X., Cheng H., Zhao Y., Wang Y., Li F. (2022). Portable electrochemical biosensor based on laser-induced graphene and MnO_2_ switch-bridged DNA signal amplification for sensitive detection of pesticide. Biosens. Bioelectron..

[83] Wu J., Yang Q., Li Q., Li H., Li F. (2021). Two-dimensional MnO_2_ nanozyme-mediated homogeneous electrochemical detection of organophosphate pesticides without the interference of H_2_O_2_ and color. Anal. Chem..

[84] Deng Z., Yang D., Chen Y., Liu X., Wu Q., Yin X., Wang J., Zhang D. (2023). Capture antibody imitator MnO_2_ nanozyme-based dual-signal immunochromatographic assay for rapid detection of Salmonella enteritidis. Chem. Eng. J..

[85] Pereira T.d.S., dos Santos D.M., Andre R.d.S., Correa D.S. (2024). Zein/MnO_2_ nanosheet composites integrated with a smartphone for colorimetric sensors for on-site detection of adulterants in milk. ACS Appl. Nano Mater..

[86] Mei H., Zhu X., Li Z., Jiang J., Wang H., Wang X., Zhou P. (2024). Manganese dioxide nanosheet-modulated ratiometric fluoroprobe based on carbon quantum dots from okra for selective and sensitive dichlorvos detection in foods. Food Chem..

[87] Li H., Su C., Si J., Sun C., Lu G., Yan X. (2024). Gold nanoclusters anchored manganese dioxide nanocomposites with high structural stability for sensitive detection of methyl parathion. Sens. Actuators B Chem..

[88] Tong F., Yang Z., Wang Z., Liu W., Jiang W., Zhu L., Wang L., Zheng M., Hou R., Zhou Y. (2023). Enzyme-mediated Ru@ UiO-66@ MnO_2_ NSs/thiamine-based ratiometric fluorescence sensor for visual detection of organophosphorus pesticide residues. Food Chem..

[89] Esmaelpourfarkhani M., Ramezani M., Alibolandi M., Abnous K., Taghdisi S.M. (2024). CRISPR-Cas12a-based colorimetric aptasensor for aflatoxin M1 detection based on oxidase-mimicking activity of flower-like MnO_2_ nanozymes. Talanta.

[90] Lv X., Frahat Foda M., He J., Zhou J., Cai J. (2023). Robust and Facile Label-Free Colorimetric Aptasensor for Ochratoxin A Detection Using Aptamer-Enhanced Oxidase-like Activity of MnO_2_ Nanoflowers. Food Chem..

[91] Gao Y., Gao X., Hou H., Qu Z., Li H., Du B., Tang P. (2024). Stabilizer-free MnO_2_ nanozyme with singlet-oxygen conducted high oxidase-like activity and fast catalysis capability for improving alkaline phosphatase assay. Sens. Actuators B Chem..

[92] Mohiuddin A.K., Ahmed M.S., Jeon S. (2022). Palladium doped α-MnO_2_ nanorods on graphene as an electrochemical sensor for simultaneous determination of dopamine and paracetamol. Appl. Surf. Sci..

[93] Wu L., Wang X., Wu X., Xu S., Liu M., Cao X., Tang T., Huang X., Huang H. (2022). MnO_2_ nanozyme-mediated CRISPR-Cas12a system for the detection of SARS-CoV-2. ACS Appl. Mater. Interfaces.

[94] Hu S., Yang C., Li Y., Luo Q., Luo H. (2022). Nanozyme sensor array based on manganese dioxide for the distinction between multiple amyloid β peptides and their dynamic aggregation process. Biosens. Bioelectron..

[95] Li J., Lai W., Ma C., Zhao C., Li P., Jiang M., Wang M., Chen S., Hong C. (2022). MnO_2_ nanosheet/polydopamine double-quenching Ru (bpy)_3_^2+^@TMU-3 electrochemiluminescence for ultrasensitive immunosensing of alpha-fetoprotein. ACS Appl. Nano Mater..

[96] Hu K., Chen X., Song X., Wu Y., Huang K., Chen P. (2024). Carbon dots and MnO_2_ nanosheet nanocomposites sensing platform for sensitive detection of oxalate in urine samples of urolithiasis patients. Talanta.

[97] Deng R., Xie X., Vendrell M., Chang Y.-T., Liu X. (2011). Intracellular glutathione detection using MnO_2_-nanosheet-modified upconversion nanoparticles. J. Am. Chem. Soc..

[98] Xiao T., Sun J., Zhao J., Wang S., Liu G., Yang X. (2018). FRET effect between fluorescent polydopamine nanoparticles and MnO_2_ nanosheets and its application for sensitive sensing of alkaline phosphatase. ACS Appl. Mater. Interfaces.

[99] Anju S.M., Varghese S., Merin K.A., Shkhair A.I., Rajeevan G., Indongo G., George S. (2024). The fluorescence turn-on detection of cardiac troponin T via folic acid-capped gold nanoclusters/manganese dioxide nanosheets. Sens. Actuators B Chem..

[100] Li J., Wei Y.-Y., Liu X.-P., Xu Z.-R. (2022). Plasmonic photothermal biosensor for visual detection of tyrosinase and dopamine based on manganese dioxide nanosheets-mediated etching of gold nanorods. Sens. Actuators B Chem..

[101] Lama S., Subedi S., Ramesh S., Shin K., Lee Y.-J., Kim J.-H. (2022). Synthesis and characterization of MnO_2_@cellulose and Polypyrrole-Decorated MnO_2_@cellulose for the detection of chemical warfare agent simulant. Materials.

[102] Meng A., Hong X., Zhang Y., Liu W., Zhang Z., Sheng L., Li Z. (2023). A free-standing flexible sensor MnO_2_–Co/rGO-CNT for effective electrochemical hydrogen peroxide sensing and real-time cancer biomarker assaying. Ceram. Int..

[103] Ma E., Wang K., Gou Y., Luo W., Wang H. (2025). Dual-Responsive MnO_2_-Loaded Carbonaceous Nanobottle Motors Fabricated by Interfacial Superassembly for On-The-Fly MicroRNA Sensing. Small.

[104] Li Z., Zhang X., Cheng H., Gu Z., He C., Wang J., Shi X., Na W., Li T., Tian L. (2024). Ru induced charge redistribution of MnO_2_ spheric nanozyme boosted hydrogen sulfide selective detection and cystathionine γ-lyase activity sensitive evaluation. Chem. Eng. J..

[105] Wang D., Meng Y.-T., Zhang Y., Wang Q., Lu W.-J., Shuang S.-M., Dong C. (2022). A specific discriminating GSH from Cys/Hcy fluorescence nanosensor: The carbon dots-MnO_2_ nanocomposites. Sens. Actuators B Chem..

[106] Li Z., Yu D., Xie J., Tian F., Lei D., Wang C. (2024). The lithium storage mechanism of zero-strain anode materials with ultralong cycle lives. ACS Appl. Mater. Interfaces.

[107] Gui J., Fan Y., Chen H., Liu F., Huang S., Wei M., Zhu X., Zhang Y., Liu M., Yao S. (2024). Redox-induced multisignal sensing based on fluorescent polydopamine nanoparticles and MnO_2_ nanozyme for the detection and differentiation of reducing substances. ACS Appl. Nano Mater..

